# NFкB is a critical transcriptional regulator of atypical cadherin FAT1 in glioma

**DOI:** 10.1186/s12885-019-6435-1

**Published:** 2020-01-28

**Authors:** Chitrangda Srivastava, Khushboo Irshad, Yakhlesh Gupta, Chitra Sarkar, Ashish Suri, Parthaprasad Chattopadhyay, Subrata Sinha, Kunzang Chosdol

**Affiliations:** 1 Department of Biochemistry, All India Institute of Medical Sciences, -110029, New Delhi, India; 2 Present address: Cell Biology, National Institute of Environmental Health Sciences, National Institutes of Health, Durham, NC 27709 USA; 3 Department of Pathology, All India Institute of Medical Sciences, New Delhi, India; 4 Department of Neurosurgery, All India Institute of Medical Sciences, New Delhi, India

**Keywords:** FAT1, NFкB (RelA), Glioblastoma, Tumor, Promoter

## Abstract

**Background:**

Overexpression of FAT1 gene and its oncogenic effects have been reported in several cancers. Previously, we have documented upregulation of FAT1 gene in glioblastoma (GBM) tumors which was found to increase the expression of proinflammatory markers, HIF-1α, stemness genes and EMT markers in glioma cells. Here, we reveal NFкB (RelA)/RelA/p65 as the transcriptional regulator of FAT1 gene in GBM cells.

**Methods:**

*In-silico* analysis of FAT1 gene promoter was performed using online bioinformatics tool Promo alggen (Transfac 8.3) to identify putative transcription factor(s) binding motifs. A 4.0 kb FAT1 promoter (− 3220 bp to + 848 bp w.r.t. TSS + 1) was cloned into promoter less pGL3Basic reporter vector. Characterization of FAT1 promoter for transcriptional regulation was performed by in-vitro functional assays using promoter deletion constructs, site directed mutagenesis and ChIP in GBM cells.

**Results:**

Expression levels of NFкB (RelA) and FAT1 were found to be increased and positively correlated in GBM tumors (*n* = 16), REMBRANDT GBM-database (*n* = 214) and TCGA GBM-database (*n* = 153). In addition to glioma, positive correlation between NFкB (RelA) and FAT1 expression was also observed in other tumors like pancreatic, hepatocellular, lung and stomach cancers (data extracted from TCGA tumor data). A 4.0 kb FAT1-promoter-construct [− 3220 bp/+ 848 bp, transcription start site (TSS) + 1, having 17 NFкB (RelA) motifs] showed high FAT1 promoter luciferase-activity in GBM cells (U87MG/A172/U373MG). FAT1 promoter deletion-construct pGL3F1 [− 200 bp/+ 848 bp, with 3-NFкB (RelA)-motifs] showed the highest promoter activity. Exposure of GBM cells to known NFкB (RelA)-activators [severe-hypoxia/TNF-α/ectopic-NFкB (RelA) + IKBK vectors] led to increased pGL3F1-promoter activity and increased endogenous-FAT1 expression. Conversely, siRNA-mediated NFкB (RelA) knockdown led to decreased pGL3F1-promoter activity and decreased endogenous-FAT1 expression. Deletion of NFкB (RelA)-motif at − 90 bp/− 80 bp [pGL3F1δ1-construct] showed significant decrease in promoter activity. Site directed mutagenesis at -90 bp/− 80 bp and ChIP assay for endogenous-NFкB (RelA) confirmed the importance of this motif in FAT1 expression regulation. Significant reduction in the migration, invasion as well as colony forming capacity of the U87MG glioma cells was observed on siRNA-mediated knockdown of NFкB (RelA).

**Conclusion:**

Since FAT1 and NFкB (RelA) are independently known to promote pro-tumorigenic inflammation and upregulate the expression of HIF-1α/EMT/stemness in tumors, targeting the NFкB (RelA)-FAT1 axis may attenuate an important tumor-promoting pathway in GBM. This may also be applicable to other tumors.

## Background

Glioblastoma (GBM) is the most aggressive form of glioma, resistant to almost all available treatment modalities [1–4]. The molecular alterations in GBM are well-characterized and the involvement of many oncogenic pathways and molecules including NFкB-pathway are known [1, 5–9]. NFкB is an important signaling molecule, upregulating pro-inflammatory and hypoxic responses that contribute to tumor pathology and resistance to therapy [8–10]. NFкB activation is known to promote tumor progression through mechanisms such as cell proliferation, apoptosis, angiogenesis, tumor metastasis, proinflammatory response, metabolic reprogramming etc. [10, 11]. High constitutive NFкB activity is the characteristic of GBM [9, 10, 12, 13] and NFкB has been evaluated as a potential target for therapeutic intervention in GBM [12, 14]. NFкB is a known transcription factor, upregulating the expression of many oncogenic and pro-inflammatory molecules having crucial roles in the aggressive progression of different tumors including GBM [10, 11, 15, 16].

We have earlier reported the oncogenic role of the FAT1 gene in GBM. This is via the maintenance of a pro-inflammatory microenvironment [17], regulation of HIF-1α via Akt/mTOR pathway [18] as well as by modulation of EMT/stemness molecules in hypoxic GBM [19]. FAT1, a transmembrane protein, is a non-classical proto-cadherin that has a role in developmental processes [20–22] as well as an oncogenic role in many human cancers like glioma, pancreatic cancer, leukemia, colon cancer, hepatocellular carcinoma (HCC) etc. [17–19, 23–26]. In many cancers including glioma, FAT1 is known to increase migration and invasion of tumor cells [17, 18, 27]. Upregulated expression of FAT1 gene in different tumors including GBM have been reported [17–19, 23, 25, 26] but till date, its upstream transcriptional regulation remains undefined.

In this study, in order to identify novel transcription factor(s) regulating FAT1 expression, we performed *in-silico* analysis of the FAT1 promoter (4.0 kb) using online bioinformatics tools i.e. Promo alggen (Transfac 8.3). These showed multiple DNA binding motifs for more than 500 transcription factors (TFs), including NFкB, c-myc, Sp1, Elk-1, c-jun, c-fos, E2F. The NFкB family of transcription factors showed multiple DNA binding motifs with high probabilistic values on the FAT1 promoter. NFкB is one of the most potential and highly active transcription factors in GBM and a known master regulator of inflammatory signaling. It is a critical regulator of HIF-1α, a master regulator of tumor hypoxia [28, 29]. We also observed increased expression and a positive association between NFкB (RelA) and FAT1 expression in GBM tumors. By in-vitro experiments like FAT1 promoter characterization and functional assays using GBM cell lines, we have been able to demonstrate the role of NFкB (RelA) as a potent transcriptional regulator of FAT1 expression. Hence, our study suggests an additional mechanism by which NFкB (RelA) can contribute to pro-tumorigenic microenvironment in GBM via FAT1.

## Methods

### Aim and study design

This study was aimed at identifying the transcription factor(s) regulating expression of FAT1 gene in glioma. For that, first we did *in-silico* identification of potential transcription factors binding on FAT1 promoter. Next, we did correlation analysis of the transcription factor, identified by *in-silico* analysis, and FAT1 expression in human GBM tumor samples obtained during study as well as in Rembrandt and TCGAGBM data sets. This was followed by in-vitro promoter characterization of FAT1 gene in glioma cells and functional role of NFкB (RelA) by analyzing migration/invasion and colony forming assay after siRNA mediated knockdown of NFкB (RelA) in U87MG glioma cells.

### *In-silico* analysis of FAT1 promoter

The FAT1 gene is located on chromosome 4q35.2. The transcription start site (TSS) of FAT1 gene was identified by aligning 5′ upstream FAT1 transcript (Acc. No. NM_005245) with the human genome sequence using NCBI-BLAST tool. The online bioinformatics tool, PROMO software; (http://alggen.lsi.upc.es/cgi-bin/promov3/promo/promoinit.cgi?dirDB=TF8.3) was used to identify motifs for FAT1 transcriptional regulatory molecule(s) on the FAT1 promoter.

### GBM-tumor samples

Surgically resected GBM samples (*n* = 16) were collected from Department of Neurosurgery/Neuropathology, AIIMS, New Delhi after approval by Institute Ethics-Committee, AIIMS, New Delhi (Ref.No IESC/T-416/01.11.2013). The histopathological diagnosis of the tumors was done by Prof Chitra Sarkar, Department of Pathology, AIIMS, New Delhi. Normal human brain total-RNA (Clontech, CA, USA) was used as control. REMBRANDT-GBM database (Affymetrix HG U133 v2.0 Plus) was accessed at http://caintegratorinfo.nci.nih.gov/rembrandt and analyzed for the expression of NFкB (RelA) and FAT1 genes in the available 214 GBM cases. In addition, TCGA expression data was also obtained from the open database available at http://www.proteinatlas.org for NFкB (RelA) and FAT1 expression in GBM as well as other tumors like pancreatic, hepatocellular, stomach and lung cancers. Gene expression data of RNA seq analyses was obtained in the form of FPKM values (data not normalized with respect to normal tissues) which was merged for NFкB (RelA) and FAT1 values and then subjected to Spearman’s correlation analysis using GraphPad Prism Version 5.00.

### Cell culture

Glioma cell lines (U87MG/A172/U373MG) were procured from ATCC (USA) and maintained in DMEM (Dulbecco’s Modified Eagle’s Medium, Hyclone), supplemented with 10% (v/v) FCS (fetal calf serum, Gibco), 3.7 g/l sodium bicarbonate and ciprofloxacin 10 μg/ml and 5% CO_2_ at 37 °C. The cells were cultured in 25 cm^2^ canted neck, vented cap tissue culture flasks, 6-well, 24-well and 96-well plates in airtight chambers. Normoxic (20% O_2_ and 5% CO_2_) conditions were maintained using vacuum pump and gas proportionator (Anoxomat), which adjust the oxygen concentration by evacuating air and replacing it with nitrogen.

### PCR amplification and cloning of FAT1-promoter

Genomic DNA was isolated from healthy human PBMCs using Qiagen kit (Germany), quantified and used to PCR amplify ~ 4.0 kb FAT1 promoter using specific primers (sense and antisense primers; Additional file 1: Table S1) flanked with MluI and NheI sites for cloning purpose. Hot-start PCR was performed using pfu-Taq polymerase in the presence of 5% DMSO, the amplified product of a 4.0 kb FAT1 promoter sequence (− 3220 bp to + 848 bp w.r.t. TSS + 1) was subjected to agarose gel electrophoresis, the amplicon was excised from the gel, purified and cloned in promoter less pGL3Basic reporter vector (Promega, promoter less vector). The vector with 4.0 kb cloned promoter was named pGL3F4. The pGL3F4 was transfected in DH5α and plated. One of the positive colonies was confirmed by double digestion with MluI-NheI as well as with KpnI restriction enzyme to confirm the 5′ to 3′ directionality of insert. The confirmation of the cloned 4.0 kb FAT1 promoter (pGL3F4) was done by sequencing and DNA-BLAST analysis. The 4.0 kb FAT1 promoter was also analyzed forGC content and TATA/CAAT Box using Eukaryotic promoter database (EDP; http://epd.vital-it.ch/index.php).

### Sequential deletion of 4.0 kb FAT1 promoter to generate promoter deletion constructs

The FAT1 promoter deletion constructs were generated by sequentially deleting the NFкB (RelA) motifs from the 5′ and the 3′ ends of the full length 4.0 kb promoter construct pGL3F4 [− 3220 bp to + 848 bp w.r.t. TSS] having 17-NFкB (RelA) binding motifs. The details of the FAT1-promoter constructs, number of NFкB (RelA) motifs present in each constructs and the sequences of the primers used are given in Additional file 1: Table S1 and Table S2.

By sequential deletion from 5′ end of the 4.0 kb promoter we generated (i) a promoter of 3.1 kb size [− 2260 bp to + 848 bp TSS] having 13-NFкB (RelA) binding motifs; (ii) a promoter of 2.0 kb size [− 1230 bp to + 848 bp w.r.t. TSS] with 10-NFкB (RelA) binding motifs; and (iii) a promoter of 1.0 kb size [− 200 bp to + 848 bp w.r.t. TSS] with 3-NFкB (RelA) binding motifs; by PCR amplification using specific primer pairs (Additional file 1: Table S1). The constructs were named as pGL3F3 (3.1 kb), pGL3F2 (2.0 kb) and pGL3F1 (1.0 kb), respectively. Further, from pGL3F2 (2.0 kb promoter) two more deletion constructs were generated viz. pGL3F2δ1of 1.3 kb [− 530 bp to + 848 bp w.r.t. TSS] having 6-NFкB (RelA) motifs; and pGL3F2δ2 of 1.1 kb [− 280 bp to + 848 bp w.r.t. TSS] having 3-NFкB (RelA) motifs. And, from pGL3F1 (1.0 kb) two more deletion constructs were generated by 5′ and 3′ deletion to generate (i) pGL3F1δ1of 0.86 kb (− 20 bp to + 848 bp w.r.t. TSS) with two NFкB (RelA) motifs at + 347 bp/+ 356 bp and + 360 bp/+ 368 bp and (ii) pGL3F1δ2 of 0.25 kb (− 200 bp to + 50 bp w.r.t. TSS) with one NFкB (RelA) motif at -90 bp/− 80 bp. The PCR-amplified products of different promoter lengths were subjected to agarose gel electrophoresis, the amplicons were gel extracted, purified using Promega PCR-cleaning Kit (Promega, USA) following manufacturer’s protocol and cloned in Luciferase expression-vector pGL3Basic (Promega, promoter less vector) at MluI/NheI sites. The sequences of all the deletion constructs were confirmed by sequencing and blast analysis.

### Promoter activity analysis by dual-luciferase assay

U87MG, A172 and U373MG cells were cultured and plated in six-well plates. After overnight growth, the cells were transfected with the FAT1 promoter constructs using lipofectamine 2000™ reagent (Invitrogen, USA). After 48 h of transfection, Luciferase activity was analysed by Dual-luciferase assay kit (Promega, USA) following manufacturer’s protocol.

### Overexpression and induction of NFкB (RelA)

#### Severe-hypoxia treatment

Glioma cells were exposed to severe hypoxia (0.2% O_2_) and normoxia (20% O_2_) using Anoxomat (Mart Chamber, Netherlands). Cells were transfected with pGL3F1 and exposed to severe hypoxia (0.2% O_2_) for 48 h, and lysates were prepared for promoter-activity analysis.

#### Recombinant-human TNFα treatment

Glioma cells were treated with recombinant human TNFα (100 ng/ml) (Biolegend; Cat no. 570108) and harvested after 24 h for mRNA expression and promoter-activity analysis in pGL3F1-transfected cells.

#### NFкB (RelA)-overexpression vectors

Glioma cells were transfected with NFкB (RelA)-expression vectors (RelA and IKBK/IKK vectors; eBABEpuro, kindly gifted by Prof. Soumen Basak, NII, New Delhi). The cells were harvested after 48 h of transfection for mRNA expression and promoter-activity analysis in pGL3F1 co-transfected cells.

### Knockdown of NFкB (RelA) expression

Glioma cells were transiently transfected with 100 μM of NFкB (RelA)-specific siRNA (ON-TARGETplus Human RelA siRNA-SMARTpool, Dharmacon) and 100 μM control-siRNA (ON-TARGETplus Non-targeting Pool) for 48 h followed by mRNA expression and promoter-activity analysis in pGL3F1 co-transfected cells. Cell migration/invasion assay and anchorage-independent growth assays were performed after knockdown of NFкB (RelA) following the methods previously published [19].

### Expression analysis by qPCR

Total RNA from treated and respective control cells was isolated using TRI reagent (Sigma, St Louis, MO). RNA quantification was done using spectrophotometer by measuring absorbance at 260 and 280 nm. Total RNA (1 μg) was used for reverse transcription using Revert Aid M-Mul-V Reverse transcriptase (MBI Fermentas, USA) and random decamers. For mRNA expression analysis, real time PCR was performed on Corbett Rotor Gene Q 6000 PCR machine. The details of primers used are listed in Additional file 1: Table S3.

### Western blot analysis

U87MG cells were transfected with 1 μg pBABE-RelA vector/control vector or with 100 μM NFкB (RelA)-specific siRNA/control-siRNA using lipofectamine 2000™ reagent (Invitrogen, USA) following manufacturer’s protocol. After 48 h of transfection, whole cell lysates were prepared using RIPA buffer (Thermo Fisher Scientific) and protease inhibitor cocktail (Sigma-Aldrich). 40 μg of the lysates were loaded in gradient SDS-PAGE gel (6–12%), subjected to gel electrophoresis in Western blot apparatus (Mini-PROTEAN Biorad gel apparatus) and transferred on to PVDF membrane. Primary antibodies used were: FAT1 (rabbit polyclonal, 1:500, Novus Biologicals) and β-actin (mouse monoclonal, 1:5000, Abkine). Densitometric analysis was done for the quantification of protein bands on the blot using ImageJ.

### Site directed mutagenesis (SDM)

Site directed mutagenesis was done for the promoter reporter to elucidate the regulatory motif in FAT1 promoter by QuikChange II Site-Directed Mutagenesis Kit, according to the manufacturer’s protocol. Primer details are given in Additional file 1: Table S3. Annealing temperature of the SDM primers was standardized. pGL3F1 with the wild type NFкB (RelA) site was taken as the parental plasmid to amplify mutated construct using SDM primers (pGL3F1mtRelA) and confirmed by digestion of plasmid with DpnI restriction enzyme followed by gel electrophoresis. The plasmid was transformed and further confirmed by EcoRI digestion. The wild type vector (pGL3F1) and the mutated vector (pGL3F1mtRelA) were subjected to EcoRI digestion followed by gel electrophoresis. Confirmation of the mutated NFкB (RelA) sequence was done by sequencing of the wild type and the mutated vectors.

### Chromatin-immunoprecipitation (ChIP)

U87MG cells were plated for 24 h and harvested for ChIP assay. Cells were fixed with 4% paraformaldehyde followed by sonication (1.5 cycle, 70% amplitude) to shear the gDNA. Sonicated lysate was pulled down with specific antibodies [Anti RelA Antibody, Anti pol-II (Positive control) and IgG (Negative control)]. Reverse cross-linking of DNA and protein was done for the PCR amplification using specific primers. Gel electrophoresis analysis confirmed the presence of ChIP product (FAT1) at 190 bp. ChIP assays were performed using ImprintTM Chromatin Immunoprecipitation Kit (Sigma-Aldrich, St. Louis, USA) according to the manufacturer’s protocol. ChIP specific primers (ChIPF and ChIPR) were used to amplify FAT1 promoter region containing NFkB (RelA) transcription binding site (Additional file 1: Table S3).

### Transwell migration and invasion assay

U87MG cells were harvested 48 h post-siRelA/siControl transfection, resuspended in serum-free medium and plated on top of transwell inserts (8 μm pore size) in triplicate wells in 24-well plates. For invasion assay, 3 × 10^4^ cells were seeded on inserts coated with matrigel, while for migration assay, 1 × 10^4^ cells were seeded on uncoated inserts. Cells were allowed to migrate (for 24 h) and to invade (for 48 h) across transwell towards serum-containing medium at 37 °C. Cells inside the upper chamber were removed using cotton swabs. Migrated/invaded cells on the lower membrane surface were fixed in 4% paraformaldehyde, stained with DAPI, and counted microscopically at 20x magnification in five non-overlapping fields per well.

### Anchorage-independent colony formation assay

In order to check the clonogenic ability of U87MG, cells were harvested 48 h post-siRelA/siControl transfection. A total of 15,000 cells/well were suspended in 0.3% LMP agarose (Invitrogen) in DMEM supplemented with 20% FBS and seeded on top of 0.6% agarose in 6-well plates in triplicates. 500 μl of complete medium was added on top of the agarose layer twice a week. After 22 days in culture, the colonies were stained using 0.2% crystal violet. Mean number of colonies with ≥30 μm diameter was counted microscopically at 4x magnification in ten non-overlapping fields per well.

### Statistical analysis

Parametric data results were expressed as means±S.E. (standard error) and comparisons were done by Student’s *t*-test. For non-parametric data, results were expressed as mean ± SEM (standard error of mean). A *p*-value ≤0.05 was considered as statistically significant. Analysis of the GBM tumor data was done by Spearman’s Rank Correlation Coefficient using GraphPad Prism Version 5.00.

## Results

### *In-silico* characterization of FAT1 promoter identifies multiple NFкB (RelA) binding motifs

To identify the binding motifs on the FAT1 promoter, first of all, the transcription start site (TSS) of FAT1 gene was identified by aligning 193 bp of 5′ upstream FAT1 transcript (Acc. No. NM_005245) with the human genome using NCBI BLAST tool, which showed 100% complementarily to the FAT1 gene (Accession number-NC_000004.12, www.ensembl.org) on chromosome 4q35.2 (Additional file 1: Fig. S1). A 4.0 kb FAT1 promoter sequence [− 3220 bp to + 848 bp, with respect to transcription start site (w.r.t. TSS + 1)] was selected from Ensembl (Accession number-NC_000004.12) for the identification of binding motifs for FAT1 transcriptional regulatory molecule(s). Using the online bioinformatics tool, PROMO software; binding motifs for more than 500 transcription factors (TFs) were recognized with high (15%) matrix dissimilarity. Binding motifs for multiple oncogenic TFs like NFкB, c-myc, sp1, Elk-1, c-jun etc. were noted on 4.0 kb-FAT1 promoter. The full 4.0 kb FAT1 promoter-sequence with representative oncogenic TFs binding sites is depicted in Additional file 1: Fig. S2. Amongst all TFs, the NFкB family of transcription factors showed multiple binding sites (17 motifs) on FAT1 promoter with high matrix score (Additional file 1: Figs. S3a & S3b). NFкB (RelA) is one of the most potent and active transcription factor of the NFкB family known to be associated with GBM [30]. Upregulated expression of FAT1 has been earlier found to be positively correlated with GBM aggressiveness [17–19, 31]. *Our in-silico finding of multiple NFкB (RelA) binding motifs on FAT1 promoter suggests NFкB (RelA) as a potential transcription factor in regulating FAT1 expression.*

### A positive correlation between NFкB (RelA) and FAT1 expression in human GBM tumor samples

**Fig. 1:**
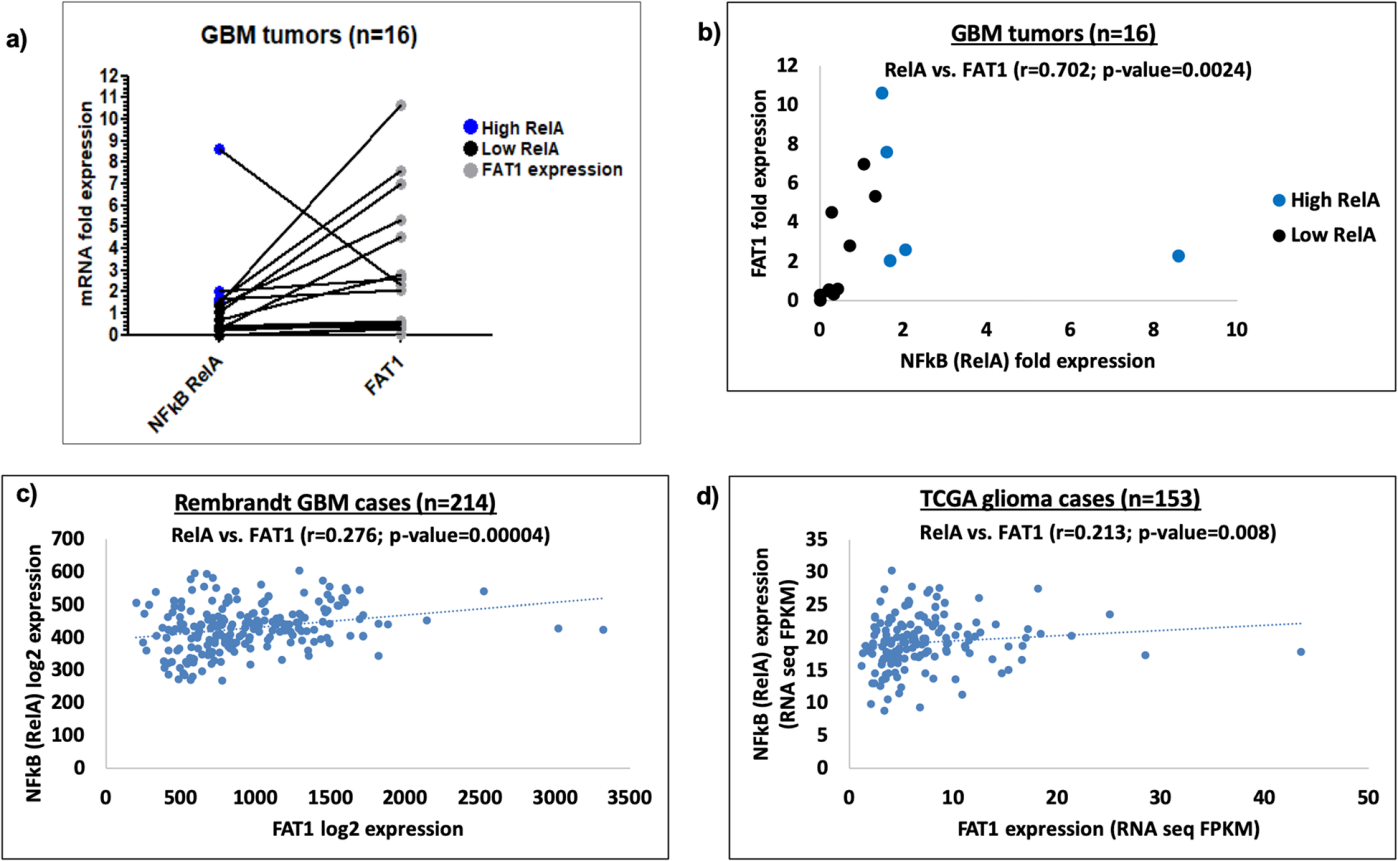
**a** Expression analysis of NFкB (RelA) and FAT1 in GBM samples**.** mRNA expression of NFкB (RelA) and FAT1 genes was analyzed in GBM tumors (*n* = 16) with respect to the levels found in human normal brain total RNA (Clontech, USA) using qPCR. Increased expression of FAT1 was found in 9 out of 16 GBM samples as compared to the normal brain. High-NFkB expressors are represented as blue dots and low-NFkB expressors as black dots. FAT1 has been depicted with grey dots. **b** Correlation analysis of NFкB (RelA) and FAT1 expression in GBM samples**.** mRNA fold expression values of NFкB (RelA) and FAT1 found in the sixteen GBM tumors were correlated using Spearman’s analysis by SPSS 11.5. A significant positive correlation was observed between NFкB (RelA) and FAT1 (*r* = 0.7, *p* = 0.002) in the studied samples. GBM samples have been displayed as high NFкB (RelA) expressors (blue dots) and low NFкB (RelA) expressors (black dots) on the basis of a cut-off value of ≥1.5 fold expression. **c** Correlation of NFкB (RelA) and FAT1 expression in Rembrandt GBM database**.** Expression values of NFкB (RelA) and FAT1 were obtained and correlated in 214 GBM cases belonging to Rembrandt database. A significant positive correlation was observed between NFкB (RelA) and FAT1 (Spearman’s ‘*r*’ = 0.276, *p* = 0.00004) in the analyzed cases. **d** Correlation of NFкB (RelA) and FAT1 expression in TCGA glioma database**.** Expression values of NFкB (RelA) and FAT1 were obtained and correlated in 153 glioma cases belonging to TCGA database. A significant positive correlation was observed between NFкB (RelA) and FAT1 (Spearman’s ‘*r*’ = 0.213, *p* = 0.008) in the analyzed cases

To test the association of our *in-silico* finding of multiple NFкB (RelA) binding sites on FAT1 promoter, the correlation of expression of FAT1 and NFкB (RelA) was analyzed in GBM tumors (*n* = 16) obtained during the study. We observed increased expression of FAT1 in over 50% of GBM samples (*n* = 9/16) as compared to the expression in normal brain (Clontech, USA) (Fig. 1a). Spearman’s correlation analysis displayed a strong positive correlation between the expression of NFкB (RelA) and FAT1 (*r* = 0.70; *p* = 0.002) (Fig. 1b). REMBRANDT GBM database (Affymetrix HG U133 v2.0 Plus), obtained from publicly available Betastasis website, was analyzed for correlation between expression of NFкB (RelA) and FAT1. A significant positive Spearman’s correlation was observed between the expression of NFкB (RelA) and FAT1 (*r* = 0.276; *p* = 0.00004) (Fig. 1c). We observed significantly increased expression of NFкB (RelA) (*p* < 0.001) and FAT1 (*p* < 0.001) in GBM tumors (*n* = 214) as compared to normal brain (*n* = 20) (data not shown). A significant upregulation of FAT1 (*n* = 107; *p* = 0.03) was observed in high NFкB (RelA) expressing GBM group as compared to low NFкB (RelA) expressing GBM group (*n* = 107), when GBM tumors were arranged in decreasing order of NFкB (RelA) expression (data not shown). We also checked the correlation of NFkB (RelA) and FAT1 expression in TCGA GBM data from the open database available on http://www.proteinatlas.org. A significant positive correlation (*r* = 0.213, *p* = 0.008) was observed between NFkB (RelA) and FAT1 expression in GBM cases (*n* = 153) (Fig. 1d). Increased FAT1 mRNA expression in GBMs and its positive correlation with NFкB (RelA) expression pointed to a potential role of NFкB (RelA) as a transcriptional regulator of FAT1. In addition, a significant positive correlation between the NFkB (RelA) and FAT1 expression was observed in non-glioma tumors like pancreatic cancer (*n* = 176) cases (*r* = 0.307, *p* = 0.00003); hepatocellular cancer (*n* = 365) cases (*r* = 0.238, *p* = 0.000004); stomach cancer (*n* = 354) cases (*r* = 0.158, *p* = 0.003) and lung cancer (*n* = 994) cases (*r* = 0.126, *p* < 0.0001) (Additional file 1: Figure S4). The oncogenic role of FAT1 has been reported in these above cancers [25, 26, 32] in addition to GBM [17–19]. *Hence, the positive correlation between NFkB (RelA) and FAT1 expression extends to other tumors and is not exclusive to GBM.*

### Characterization of the FAT1 promoter identifies regulatory NFкB (RelA) motif(s) in GBM cells

**Fig. 2:**
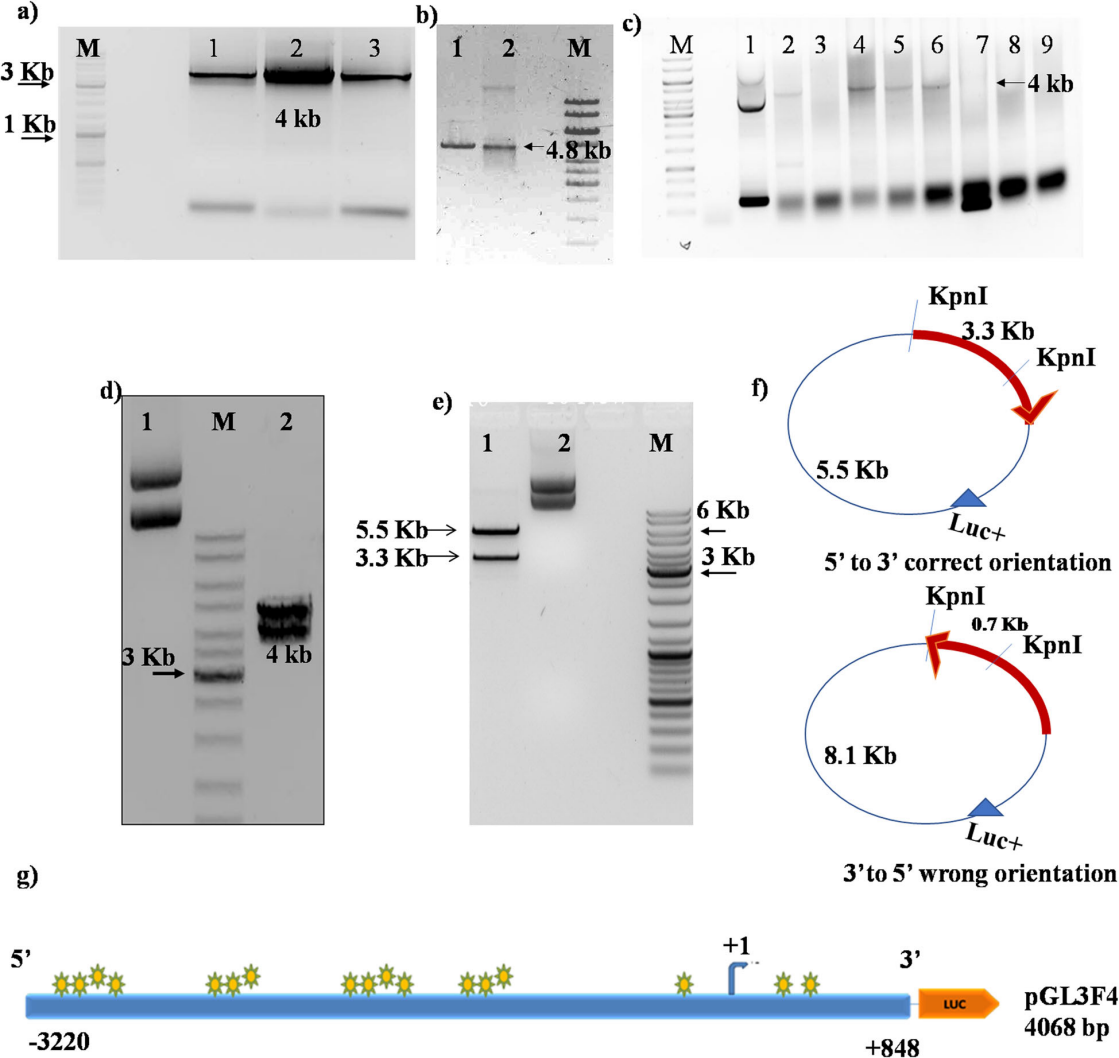
Cloning of FAT promoter region in pGL3Basic: **a** FAT1 promoter (4.0Kb) was amplified by FAT1 gene specific primers flanked with restriction enzyme sites (MluI and NheI) from gDNA (human PBMCs). 4.0 kb PCR product was observed in gel electrophoresis. Lanes 1, 2 and 3 represent amplified FAT1 promoter at 4.0 kb, Lane M: Marker. **b** pGL3Basic (promoterless vector) digested with MluI and NheI restriction enzymes and analyzed by gel electrophoresis. Lane 1: Digested (4.8 kb linearized pGL3B), Lane 2: Undigested pGL3B. **c** Colony PCR was done to confirm the ligation of insert (FAT1 promoter) in pGL3Basic. FAT1 PCR product was eluted from gel electrophoresis followed by restriction digestion with MluI and NheI, ligated with digested pGL3Basic and transformation was done in E.coli strain (DH5 alpha). 8 colonies were picked and confirmed by colony PCR using vector specific primers (RV-GL). Lane1: positive control pGL3Control (SV40 promoter) (250 bp PCR product), Lane 2, 4, 5 and 6: Four colonies were found to be positive having 4.0 kb insert amplified product. **d** Positive colony was confirmed by double digestion with MluI-NheI. Lane1: undigested pGL3F4 vector and lane 2: Digested pGL3F4; released 4.0 kb (insert) and 4.8 kb vector backbone. **e** Cloned FAT1 promoter (pGL3F4 construct) digested with KpnI restriction enzyme to confirm the 5′ to 3′ directionality of insert. Lane 1: Digested pGL3F4 with KpnI released 3.3 kb insert and 5.5 kb vector backbone plus part of the insert and lane 2: Undigested pGL3F4. **f** A representative diagram of FAT1 construct (pGL3F4) showing position of KpnI site on vector (pGL3B) (at + 5 position) and insert [FAT1 promoter at + 105 bp w.r.t. Transcription start site (+ 1)]. **g** A representative diagram of the full length 4.0 kb FAT1 promoter construct (pGL3F4) that was used in cloning

The identification of multiple NFкB (RelA) binding motifs on FAT1 promoter and a positive correlation observed between FAT1 and NFкB (RelA) expression in GBM tumors provided a strong rationale for studying the transcriptional regulation of FAT1 by NFкB (RelA). To characterize the regulatory role of NFкB (RelA) as potential transcription factor, a 4.0 kb 5′ upstream sequence of FAT1 gene (− 3220 bp to + 848 bp w.r.t. TSS + 1) having seventeen (17) NFкB (RelA) binding motifs (Additional file 1: Figures S3a & S3b) was cloned in pGL3Basic reporter vector (pGL3F4) (Fig. 2). The cloned 4.0 kb promoter sequence was confirmed by sequencing and BLAST analysis (Additional file 1: Figure S5). The FAT1 promoter has been found to have high GC content in the core promoter but lacks the TATA Box and CAAT Box in its promoter (Additional file 1: Figure S6).

**Fig. 3:**
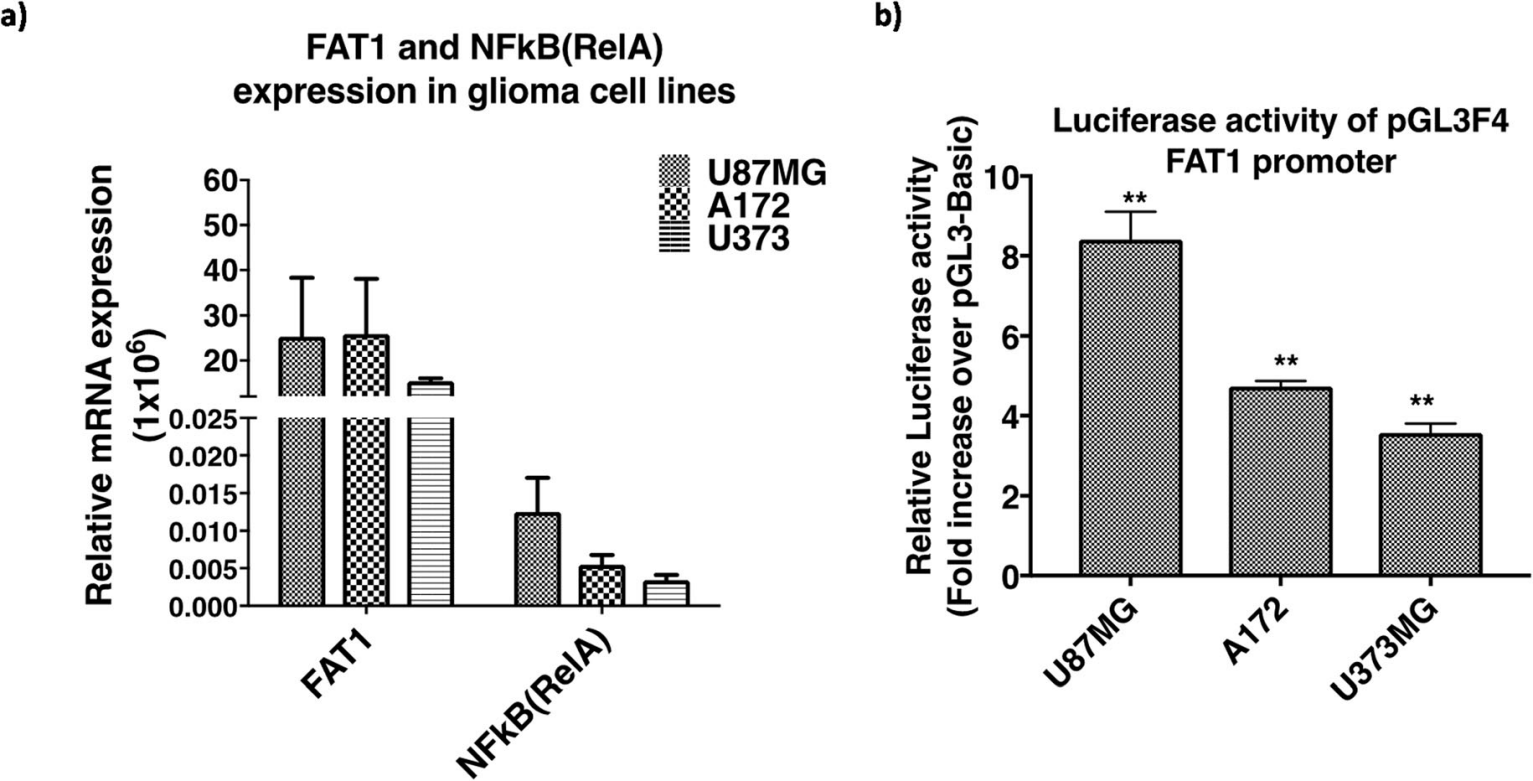
**a** Expression of FAT1 and NFкB (RelA) in glioma cell lines. Increased relative mRNA expression of FAT1 & NFкB (RelA) in U87MG, A172 and U373MG was observed. **b** Luciferase activity of full length 4.0 kb FAT1 promoter (pGL3F4) in glioma cell lines. Glioma cell lines (U87MG, A172 and U373MG) were transfected with pGL3F4 and pGL3B (promoterless vector). Luciferase activity was analysed by dual luciferase assay after 48 h of transfection. Significant increase in luciferase activity of pGL3F4 was found, with U87MG showing the highest luciferase activity followed by A172 and U373MG

As per earlier reports of high NFкB (RelA) [10] and FAT1 expression [17] in GBM cells, high relative mRNA expression of FAT1 and NFкB (RelA) was observed in GBM cells with the highest level in U87MG followed by A172 and U373MG (Fig. 3a). The 4.0 kb promoter construct (pGL3F4) was transfected in GBM cells (U87MG, A172 and U373MG) and luciferase activity was analysed by dual luciferase assays after 48 h of transfection. Increased luciferase activity of pGL3F4, normalized to the promoter less vector-control pGL3Basic, was observed in all GBM cells, with the highest activity in U87MG (8.41 fold ±0.70) followed by A172 (4.73 fold ±0.14) and U373MG (3.57 fold ±0.23), as compared to their respective controls (Fig. 3b).

**Fig. 4:**
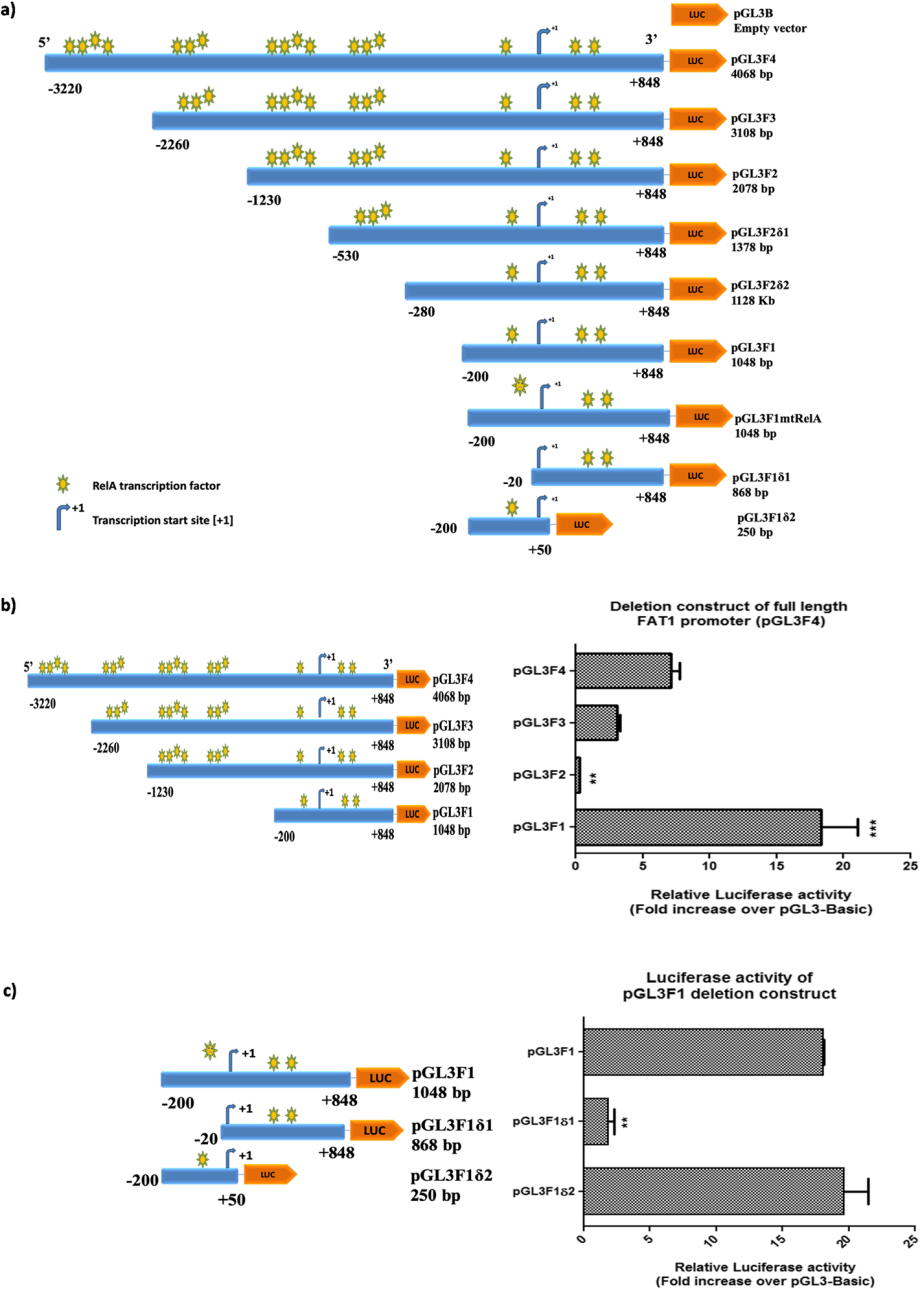
**a** A schematic diagram of FAT1 promoter constructs. 5′ and 3′ deletion constructs were generated from 4.0 kb full length FAT1 promoter (− 3220/+ 848 bp). To amplify FAT1 promoter region (4.0 kb) primers were designed (using Primer 3 software) and 5′ flanked with restriction enzymes, *MluI* in forward primer and *NheI* in reverse primer. To amplify 5′ sequentially deleted FAT1 construct, forward primers were designed at the corresponding sites (at -2260 bp, − 1230 bp, − 530 bp, − 280 bp, − 200 bp, − 20 bp) of the respective FAT1 constructs [− 3220 bp/+ 848 bp w.r.t. TSS (+ 1)] while the reverse primer (R) (at + 848 bp) was constant. To amplify 3′ deletion FAT1 construct, reverse primer was designed at corresponding site (at + 50 bp) of respective FAT1 construct and forward primer (at -200 bp) was paired. The FAT1 promoter inserts (F4, F3, F2, F2δ1, F2δ2, F1, F1δ1 and F1δ2) display TSS (+ 1), size of insert, 5′ upstream and 3′ downstream region of FAT1 promoter and yellow-green stars showing binding sites of NFkB (RelA) on each construct. **b** Luciferase activity of 5′ deletion constructs (pGL3F3, pGL3F2 and pGL3F1) of FAT1 promoter (pGL3F4). U87MG cells were transfected with pGL3F4 (4.0 kb) and its 5’deletion constructs [pGL3F3 (3.1 kb), pGL3F2 (2.0 kb) and pGL3F1 (1.0 kb)] for 48 h followed by dual luciferase assay. pGL3F1 showed the highest luciferase activity followed by pGL3F4, pGL3F3 and pGL3F2 as compared to pGL3B. **c** Luciferase activity of 5′ and 3′ deletion constructs of FAT1 promoter construct (pGL3F1): FAT1 promoter constructs [pGL3F1δ1 (0.86 kb) and pGL3F1δ2 (0.25 kb)] and pGL3F1 were transfected in U87MG; and luciferase activity was analyzed after 48 h. A comparable luciferase activity was observed in pGL3F1 and pGL3F1δ2. A significant decrease in the luciferase activity was observed in pGL3F1δ1 (4.43 fold ±0.61) as compared to pGL3F1. Bar graph represents fold luciferase activity of FAT1 promoter constructs (normalized to pGL3Basic and internal control pRLTK). Values are mean ± SE of at least three independent experiments performed in triplicates. Statistical analysis was performed using a paired two-tailed Student’s *t*-test. *P*-value < 0.05 was taken as significant (*p < 0.05, **p < 0.01, ***p < 0.001). Generated constructs were cloned and confirmed by sequencing and BLAST analysis

To characterize the potential NFкB (RelA) motif(s) on the FAT1 promoter, important for FAT1 expression regulation, a total of seven deletion constructs were generated (Fig. 4a) by sequentially deleting the NFкB (RelA) motifs at 5′ and 3′ ends of FAT1 promoter (pGL3F4) construct. The deletion constructs pGL3F3 [with 13-NFкB (RelA) motifs], pGL3F2 [with 10-NFкB (RelA) motifs] and pGL3F1 [with 3-NFкB (RelA) motifs] were transfected in U87MG cells for 48 h. Luciferase activity was analysed along with the parent construct pGL3F4 [having 17-NFкB (RelA) motifs]. Among all promoter constructs, pGL3F1 showed the highest activity (18.29 fold ±2.76), followed by pGL3F4 (7.14 fold ±0.61), pGL3F3 (3.06 fold ±0.21) and pGL3F2 (0.29 fold ±0.02) as compared to pGL3basic empty vector (Fig. 4b). The reduced promoter activity of pGL3F2 could be due to the binding of trans-repressors as identified in our in-silico analysis. However, in this study we have focused on regulation of FAT1 expression by trans-activator [e.g. NFкB (RelA)].

To narrow down the regulatory NFкB (RelA) motif(s), we further deleted NFкB (RelA) motif(s) from 5′ and 3’ends of FAT1 promoter construct pGL3F1 with three NFкB motifs to generate (i) pGL3F1δ1 [with two NFкB (RelA) motifs] and (ii) pGL3F1δ2 with one NFкB (RelA) (Fig. 4a). The luciferase activity of these constructs (pGL3F1/pGL3F1δ1/pGL3F1δ2) was analyzed in U87MG cells. We observed comparable promoter activity of pGL3F1 and pGL3F1δ2 with 18 fold±0.61 and 19.65 fold±1.78, respectively. Interestingly, pGL3F1δ1, with deleted NFкB (RelA) site at -90 bp/− 80 bp, showed significant reduction in the promoter activity (4.43 fold ±0.61) as compared to pGL3F1 (Fig. 4c).

We also generated two more deletion constructs pGL3F2δ1 with 6-NFкB (RelA) motifs and pGL3F2δ2 with 3-NFкB (RelA) motifs from pGL3F2 construct (Fig. 4a). The promoter constructs pGL3F2δ1 and pGL3F2δ2 have shown higher luciferase activity than the parent construct pGL3F2. However, the luciferase activity observed in these constructs pGL3F2/pGL3F2δ1/pGL3F2δ2 was lower than pGL3F1 (Additional file 1: Figure S7).

*The above findings suggested that the potential FAT1 promoter region (pGL3F1) from -200bp to +848bp w.r.t. TSS(+1) and NFкB (RelA) binding motif at -90bp/-80bp might be a key regulatory site to control FAT1 expression in GBM cells.*

### NFкB (RelA) upregulates FAT1 promoter (pGL3F1 construct) activity and endogenous expression in GBM

Since the potential FAT1 promoter region is from -200 bp to +848 bp w.r.t. TSS(+1), we further validated the role of NFкB (RelA) on FAT1 promoter (pGL3F1) activity and expression of endogenous-FAT1 mRNA in U87MG cells by increasing the NFкB (RelA) levels in the cells by exposing to known NFкB (RelA) modulators.

#### Severe hypoxia (0.2% O_2_) upregulates FAT1 promoter activity and endogenous FAT1 expression

**Fig. 5:**
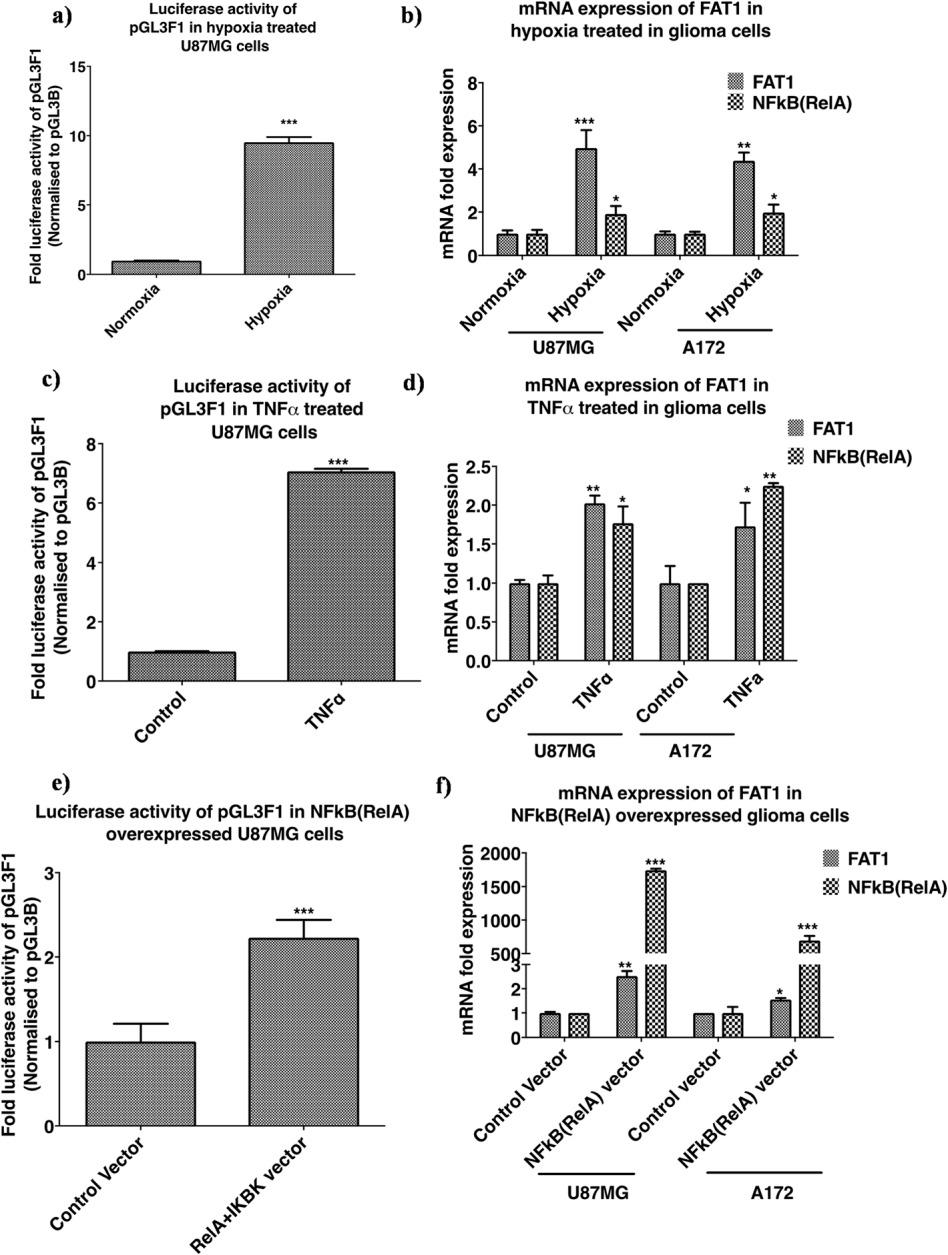
Effect of NFкB pathway activation on FAT1 promoter activity and endogenous FAT1 expression. NFкB pathway activation was done by treating GBM cells with known NFкB activators (severe hypoxia, TNFα and NFкB expression vector). **a** FAT1 promoter activity under severe hypoxia. GBM cells transfected with pGL3F1 were exposed to severe hypoxia (0.2% O_2_) and normoxia (20% O_2_) for 48 h. Luciferase activity of pGL3F1 was significantly increased under severe hypoxia as compared to normoxic control U87MG. **b** FAT1 mRNA expression under severe hypoxia. Glioma cells exposed to severe hypoxia showed significant increase in FAT1 mRNA expression along with increased NFкB (RelA) expression in both U87MG and A172 as compared to the respective normoxic cells. **c** FAT1 promoter activity in TNFα treated glioma cells. GBM cells were transfected with pGL3F1 and treated with TNFα (100 ng/ml) for 24 h followed by FAT1 promoter activity and mRNA expression analyses. Significant increase in pGL3F1 luciferase activity was observed in U87MG cells treated with TNFα as compared to the control (untreated cells). **d** FAT1 mRNA expression in TNFα treated glioma cells. Glioma cells were treated with TNFα as described above. Increased endogenous FAT1 mRNA expression in both U87MG and A172 was seen along with increased NFкB (RelA) expression, compared to the untreated control cells. **e** FAT1 promoter activity in glioma cells with overexpressed NFкB (RelA). GBM cells were co-transfected with pGL3F1 + RelA+IKBK and control vector (pGL3B + eBABE) for 48 h followed by FAT1 promoter activity and mRNA expression analyses. Significantly increased FAT1 promoter activity was observed in the cells co-transfected with RelA+ IKBK expression vector as compared to the control cells. **f** FAT1 mRNA expression in glioma cells with overexpressed NFкB (RelA). U87MG and A172 cells co-transfected with RelA+ IKBK showed increased mRNA expression of FAT1 and NFкB (RelA) expression as compared to the control cells. Bar graph represents fold luciferase activity of FAT1 promoter constructs (normalized to pGL3Basic and internal control pRLTK) and fold mRNA expression. All experiments were done in triplicates and repeated thrice. Statistical analysis was performed using a paired two-tailed Student’s *t*-test. *P*-value < 0.05 was taken as significant (*p < 0.05, **p < 0.01, ***p < 0.001)

Since hypoxia is known to positively upregulate NFкB pathway [15, 16, 33], U87MG cells transfected with FAT1 promoter construct-pGL3F1 which had the highest promoter activity were exposed to severe hypoxia (0.2% O_2_) for 48 h. The promoter activity was measured and compared to pGL3F1 transfected cells exposed to normoxia (20% O_2_). There was significant increase (9.5 fold ±0.37) in pGL3F1 activity on exposure to severe hypoxia as compared to normoxia (Fig. 5a). In addition, U87MG and A172 showed increased endogenous-FAT1 expression [4.97 fold ±0.82 and 4.38-fold ±0.38 in hypoxic cells (0.2% O_2_, 48 h) respectively] as compared to the respective normoxic cells (Fig. 5b). The increase in NFкB (RelA) transcript levels in U87MG and A172 was 1.91-fold±0.36 and 1.97-fold±0.36 respectively on severe hypoxia exposure (Fig. 5b). This suggests the positive influence of hypoxia-upregulated NFкB (RelA) on FAT1 promoter activity.

#### TNFα upregulates FAT1 promoter activity and endogenous FAT1 expression

TNFα causes phosphorylation of IKBα (Inhibitor of NFкB) followed by proteosomal degradation, leading to release of the transcription factor NFкB (RelA) and its nuclear transport [13, 30]. To check for the effect of TNFα treatment on FAT1 promoter activity, U87MG cells were transfected with FAT1 promoter (pGL3F1 construct) followed by TNFα (100 ng/ml) treatment every 24 h for 48 h followed by measurement of pGL3F1 promoter activity. There was significant increase in pGL3F1 promoter activity (7.03-fold±0.08) in cells on TNFα treatment as compared to TNFα untreated cells (Fig. 5c). U87MG and A172 cells treated with TNFα (100 ng/ml) for 24 h also showed increased endogenous FAT1 mRNA expression by 2.02-fold ±0.09 and 1.72-fold ±0.30 along with increased NFкB (RelA) expression by 1.76-fold ±0.21 and 2.24-fold ±0.03 respectively as compared to TNFα untreated control cells (Fig. 5d).

#### Exogenous expression of NFкB (RelA) upregulates FAT1 promoter activity and endogenous FAT1 expression

Next, we overexpressed NFкB (RelA) by co-transfection of RelA vector [NFкB (RelA) transcription factor- cDNA cloned in eBABE vector] and it transcriptional activator, IKBK construct (IKK/NFкB kinase- cDNA cloned in eBABE vector) (constructs were kindly gifted by Dr. Soumen Basak, NII, New-Delhi) in glioma cells. This was followed by analysis of FAT1 promoter activity and endogenous FAT1 transcript level.

U87MG cells were co-transfected with pGL3F1 + RelA+IKBK constructs or pGL3F1 + control vectors (pGL3B + eBABE vectors) and analyzed for FAT1 promoter activity 48 h post-transfection. Cells transfected with NFкB (RelA) (RelA+IKBK vector) showed significantly increased FAT1 promoter (pGL3F1) activity (2.22-fold ±0.21) as compared to control cells (transfected with empty-vectors) (Fig. 5e). We also observed increased mRNA expression of endogenous-FAT1 by 2.52-fold ±.20 and 1.55-fold±0.06 in U87MG and A172 cells respectively along with increased NFкB (RelA) level by 1745.9-fold ±21.9 and 695.0-fold ±.68.04 fold in U87MG and A172 respectively in NFкB (RelA) overexpressed cells as compared to control cells (empty vectors) (Fig. 5f).

### NFкB (RelA) abrogation downregulates FAT1 promoter activity and endogenous FAT1 expression in GBM cells

**Fig. 6:**
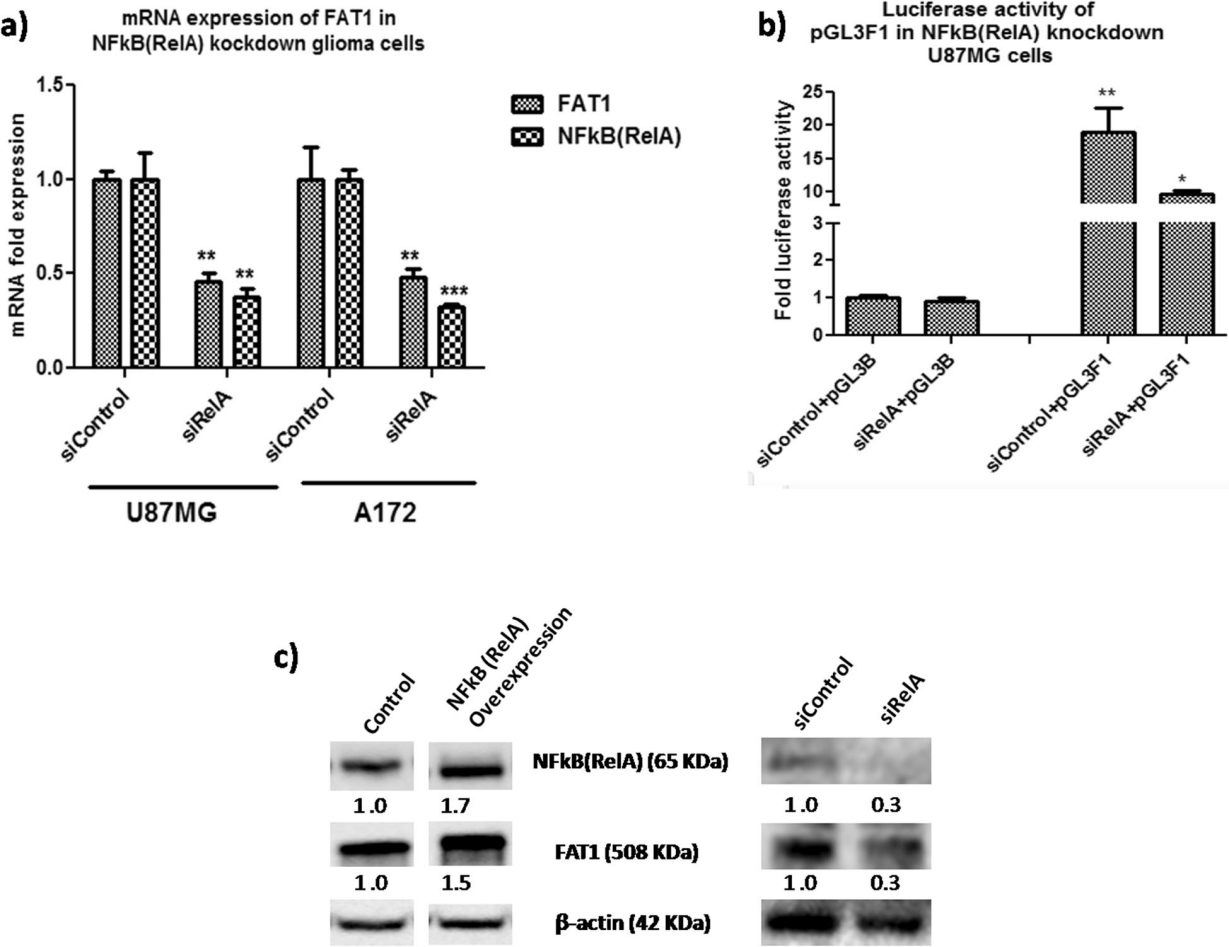
Effect of NFкB inhibition on FAT1 promoter activity and FAT1 expression in glioma cells. **a** U87MG and A172 cells were transfected with NFкB (RelA) specific siRNA (siRelA- 100 μM) and siControl (100 μM) for 48 h. Using qPCR, U87MG and A172 showed 63 and 68% knockdown of NFкB (RelA), respectively. U87MG showed decreased FAT1 mRNA expression by 55%, while in A172 FAT1 mRNA expression decreased by 52% in comparison to siControl-treated cells. **b** U87MG cells were co-transfected with NFкB (RelA) specific & control siRNA (siControl/siRelA) and pGL3B/pGL3F1 promoter constructs (pGL3F1 + siControl, pGL3F1 + siRelA, pGL3B + siControl and pGL3B + siRelA) to analyze FAT1 promoter activity after 48 h post transfection. Glioma cells transfected with pGL3F1 + siControl showed significant increase in FAT1 promoter activity (18.98 fold ±3.63) as compared to control cells (siControl+pGL3B) cells. The increased luciferase activity of pGL3F1 was significantly abrogated by siRelA as compared to siControl (from 18.98 fold ±3.63 to 9.53 fold ±0.66 luciferase activity). Bar graphs represent fold luciferase activity of FAT1 promoter constructs (normalized to pGL3Basic and internal control pRLTK) and fold mRNA expression. Values are mean ± SE of at least three independent experiments performed in triplicates. Statistical analysis was performed using a paired two-tailed Student’s *t*-test. *P*-value < 0.05 was taken as significant (*p < 0.05, **p < 0.01, ***p < 0.001). **c** Western blot analysis showed increased FAT1 protein level upon overexpression of NFкB (RelA) and decreased FAT1 protein level upon NFкB (RelA) knockdown in U87MG cells. β-actin was used as a loading control

The role of NFкB (RelA) in regulating FAT1 expression has been further validated by abrogating NFкB (RelA) level by using NFкB (RelA) specific siRNA in GBM cells. U87MG and A172 cells were transfected with 100 μM of NFкB (RelA) specific siRNA (siRelA) and control siRNA (siControl) for 48 h and observed 63 and 68% knockdown of NFкB (RelA) in U87MG and A172 respectively (Fig. 6a). On NFкB (RelA) knockdown, there was significant reduction in the expression of FAT1 in U87MG (55%-reduction) and A172 (52%-reduction). In addition, the FAT1 promoter activity (pGL3F1) was significantly (*p* ≤ 0.05) reduced in siRelA treated cells as compared to siControl treated cells, normalized to pGL3Basic (Fig. 6b). Similarly, FAT1 reduction was observed at protein level on NFкB (RelA) knockdown in U87MG cells and increased FAT1 was observed at protein level on overexpression of NFкB (RelA) in U87MG cells (Fig. 6c). *Hence, these results confirm the regulatory effect of NFкB (RelA) on FAT1 promoter activity as well as on the endogenous-FAT1 expression in GBM cells.*

### Site directed mutagenesis and chromatin Immunoprecipitation (ChIP) assay confirm the functional association of NFкB (RelA) with FAT1 promoter

**Fig. 7:**
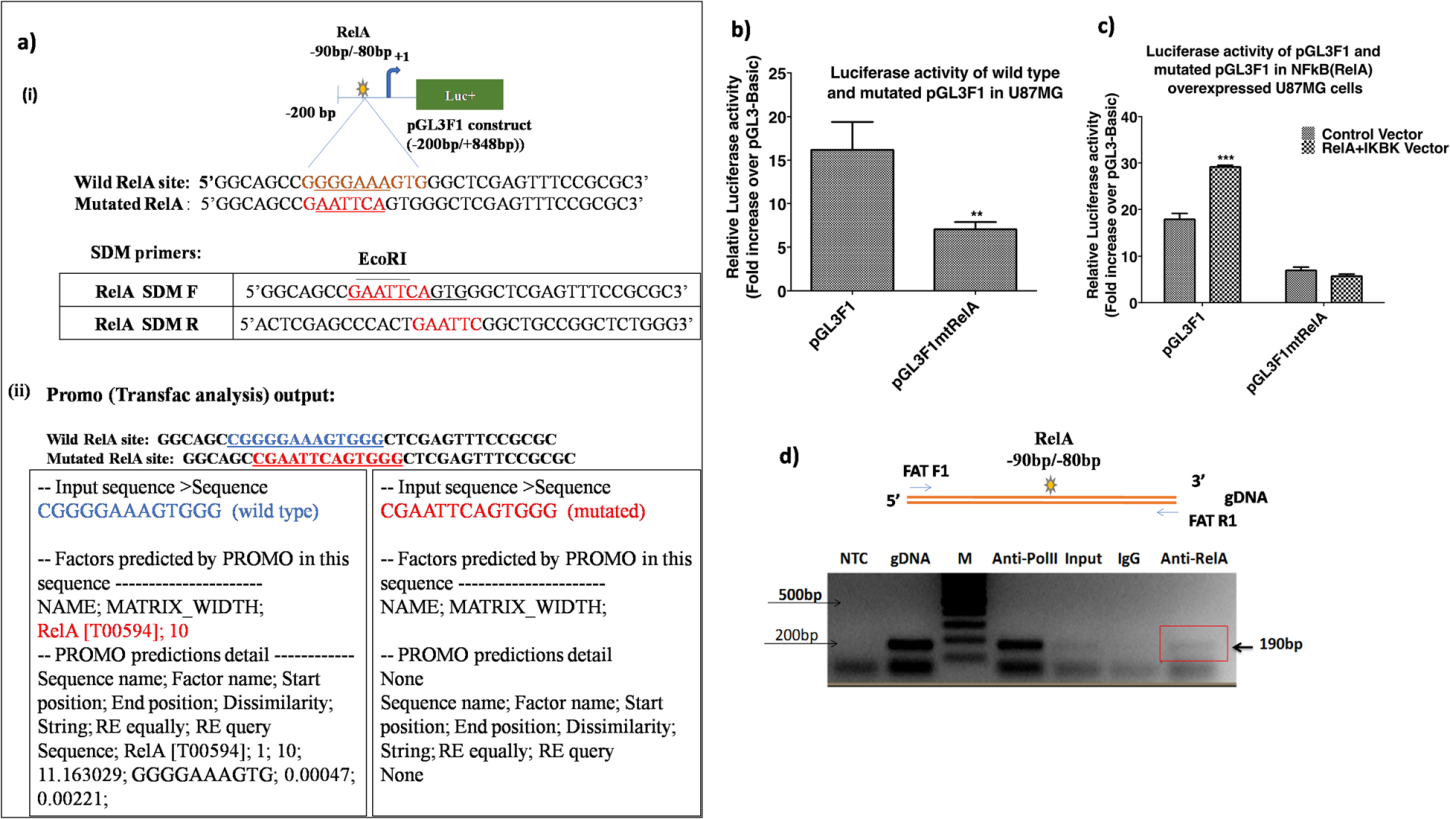
Confirmation of functional association of NFкB (RelA) on FAT1 promoter. **a i)** A representative diagram of pGL3F1 construct (− 200 bp/+ 848 bp) showing -200 bp upstream promoter sequence attached to luciferase reporter gene showing one NFkB (RelA) site (yellow green star) at − 90 bp/− 80 bp. NFkB (RelA) SDM primers are complementary to putative site of NFkB (RelA) site (at -90 bp/− 80 bp) with base (purine and pyrimidine) changes with integrated EcoRI site (5’GAATTC3’). Primer sequence of wild type and mutated NFkB (RelA) site for SDM are given. **ii)** *In-silico* validation for wild and mutated NFkB (RelA) site at -90 bp/− 80 bp was done using Promo output. NFkB (RelA) wild type sequence shows NFkB (RelA) binding site while NFkB (RelA) mutated sequence has no NFkB (RelA) binding site. **b** Effect of site directed mutagenesis of NFкB (RelA) on FAT1 promoter activity. U87MG cells were transfected with FAT1 promoter constructs, pGL3F1 and pGL3F1mtRelA. Promoter luciferase activity was analysed after 48 h. Significant decrease in luciferase activity of pGL3F1mtRelA by 56% was observed as compared to pGL3F1. **c** Effect of NFкB overexpression on promoter activity of wild type construct (pGL3F1) and mutated construct (pGL3F1mtRelA) in glioma cells. U87MG cells were co-transfected with pGL3F1 + IKBK+RelA, pGL3F1mtRelA + IKBK+RelA, pGL3F1 + eBABE, pGL3F1mtRelA + eBABE and control vector (pGL3Basic + eBABE) for 48 h. Promoter activity in pGL3F1 construct, with wild type NFкB (RelA) binding site, was increased in response to NFкB (RelA) overexpression (pGL3F1 + IKBK+RelA cells). However, promoter activity in pGL3F1mtRelA was reduced by 60% as compared to pGL3F1 construct and did not show any increase after NFкB (RelA) overexpression. Fold luciferase activity was analysed using pGL3Basic (negative control). Values are mean ± SE of three independent experiments performed in triplicates. Statistical analysis was performed using paired two-tailed Student’s *t*-test (*p < 0.05, **p < 0.01, ***p < 0.001). **d** ChIP assay. A diagrammatic representation of the ChIP product from FAT1 promoter showing NFкB (RelA) binding site at -90/− 80 bp and primers (FATF1 and FATR1) complimentary to FAT1 gene flanking the NFкB (RelA) motif is shown. Input: sonicated and reverse cross-linked whole gDNA templates U87MG, IgG (negative control): immunoprecipitated with anti-mouse IgG, Anti-RelA: immunoprecipitated with anti-RelA antibody, Anti-Pol II (positive control): immunoprecipitated with anti-Pol II antibody, gDNA: positive PCR control, NTC: no template control, M: marker (100 bp ladder). ChIP product (FAT1) of 190 bp size has been indicated

The FAT1 promoter deletion analysis of pGL3F1 suggests that NFкB (RelA) motif at -90/− 80 bp (pGL3F1δ1, with deleted NFкB (RelA) site at -90 bp/− 80 bp) may be the most potential regulatory site on FAT1 promoter (Fig. 4c). Hence, to confirm its role in FAT1 expression a site directed mutagenesis was carried out at this site of NFкB (RelA) at -90 bp/− 80 bp in pGL3F1 promoter construct (Wild RelA site: 5’GGGGAAAGTG3’: Mutated RelA site: 5’GAATTCAGTG3’). Blast analysis was done to confirm the sequence specificity of wild type (with Matrix score-11.16 for NFкB (RelA) and mutated (non binding) NFкB (RelA) binding site by Promo software (Fig. 7a). PCR amplification of mutated RelA construct (pGL3F1mtRelA) from wild type construct (pGL3F1) was done by using QuikChange II Site-Directed Mutagenesis Kit (Additional file 1: Figure S8) and confirmed by *EcoR*I digestion and sequencing (Additional file 1: Figure S9).

U87MG cells were transfected with pGL3F1 construct [wild type NFкB (RelA) site at -90 bp/− 80 bp] and pGL3F1mtRelA construct [mutated NFкB (RelA) site at -90 bp/− 80 bp] and 48 h post transfection the promoter activity was analyzed. We observed a significant decrease (*p* < 0.01) in the promoter activity in mutated pGL3F1mtRelA construct as compared to the wildtype pGL3F1 construct (Fig. 7b).

In order to confirm the specificity of NFкB (RelA) site, we also analysed the activity of both wild-type and mutated FAT1 promoters (pGL3F1 and pGL3F1mtRelA, respectively) in cells overexpressed with of NFкB (RelA) by co-transfecting RelA+IKBK expression vectors and compared with vector-control (empty vector-eBABE) treated cells. The relative luciferase activity of wild type FAT1 promoter (pGL3F1) was significantly increased (*p* < 0.01) to 29.33-fold±0.15 in NFкB (RelA) overexpressing cells (co-transfected with RelA+IKBK vectors) as compared to 18.08-fold±1.05 in control cells (i.e. eBABE vector) (Fig. 7c).

Also, promoter luciferase activity in the construct pGL3F1mtRelA with mutated NFкB (RelA) binding site was significantly decreased by 60% as compared to the pGL3F1 construct with wild type NFкB (RelA) binding site and it did not show any increase in the luciferase activity after NFкB (RelA) overexpression (IKBK+RelA vectors) (Fig. 7c).

After confirming the regulatory role of NFкB (RelA) motif -90 bp/− 80 bp, by site directed mutagenesis, ChIP assay was performed to confirm the binding of endogenous NFкB (RelA) at -90 bp/− 80 bp site on FAT1 promoter. For that, U87MG cells were plated and cultured under standard culture conditions for 24 h, cells were fixed with 4% paraformaldehyde followed by sonication (1.5 cycles, 70% amplitude) to shear the gDNA. Sonicated lysates were pulled down with specific antibodies against NFкB (RelA), pol-II (positive control) and IgG (negative control). After reverse cross-linking of pulled-down DNA and protein, PCR amplification of pulled-down DNA was done using primers (Additional file 1: Table S3) designed to amplify flanking region of NFкB (RelA) motif (at -90 bp/− 80 bp) on FAT1 promoter corresponding to 190 bp product. Gel electrophoresis analysis confirms the presence of specific ChIP product at 190 bp size (Fig. 7d), confirming the presence of NFкB (RelA) motif (at -90 bp/− 80 bp) and the cooperative binding of NFкB (RelA) to endogenous FAT1 promoter.

*Reduced promoter activity of the mutated FAT1 promoter (pGL3F1mtRelA) as well as positive ChIP assay finding of endogenous NFкB (RelA) binding to the motif [-90bp/-80bp wrt TSS(+1)] therefore, confirm the significance of transcription factor NFкB (RelA) binding to the FAT1 promoter and its role in regulating FAT1 transcription.*

### NFkB (RelA) silencing decreases cell migration, cell invasion and anchorage-independent colony formation capacity in U87MG cells

**Fig. 8:**
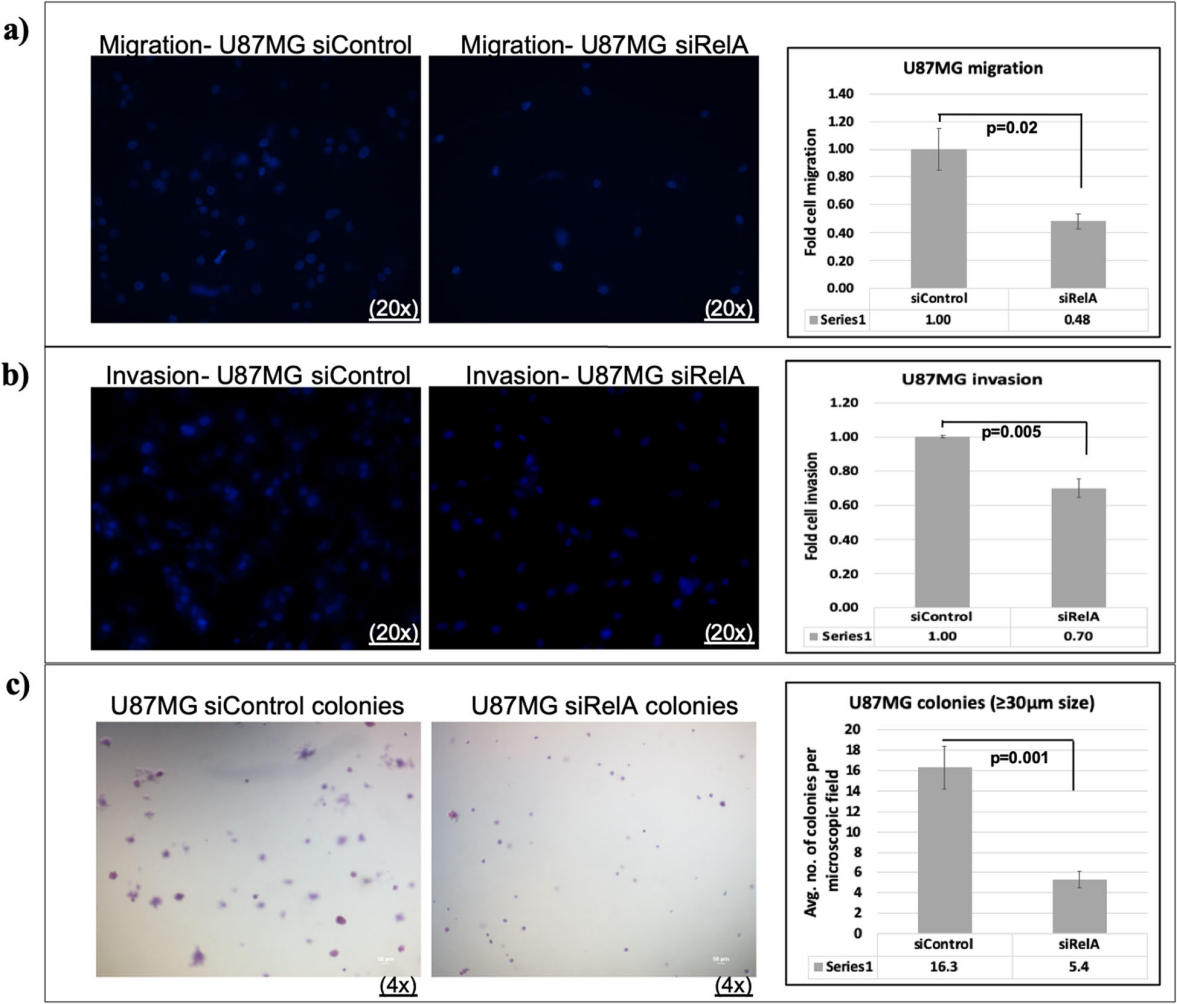
Effect of NFкB (RelA) knockdown on migration, invasion and clonogenicity of U87MG cells. Representative images (20x magnification) and quantification of (**a**) cell migration and (**b**) cell invasion in U87MG after NFкB (RelA) knockdown are shown. Transwell cell migration and invasion (matrigel) assays were performed for 24 and 48 h respectively, using U87MG cells harvested after 48 h of siRelA/siControl transfection. Cells that migrated across transwell membrane or invaded through matrigel were fixed, stained and counted. Mean of five non-overlapping fields was calculated in each of the triplicate wells. Number of cells that migrated/invaded in siRelA cells were normalized against the number of cells that migrated/invaded in siControl cells. Values in graphs are expressed as fold change. Significant decrease in migration and invasion was observed in siRelA-treated U87MG cells as compared to the siControl cells as determined by Student’s *t*-test. **c** Anchorage-independent colony formation assay. Representative micrographs (4x magnification; scale bar: 50 μm) and quantification of colonies in soft agar assay of U87MG after NFкB (RelA) knockdown are shown. U87MG cells were harvested after 48 h of siRelA/siControl transfection and 15,000 cells/well were seeded in 0.3% agarose in DMEM supplemented with 20% FBS in triplicate wells in a six-well plate. After 22 days, colonies were stained with crystal violet. Mean number of colonies (≥30 μm size) of ten non-overlapping fields was calculated in each of the triplicate wells in siRelA/siControl cells. Graph values are expressed as mean ± s.d. Significant difference was observed in siRelA-treated U87MG cells as compared to the siControl cells as determined by Student’s *t*-test

In order to confirm that the increase in migration, invasion and colony forming capacity of GBM cells mediated by FAT1 [17, 19] occurs under the transcriptional control of NFkB (RelA), we transfected U87MG cells with NFkB (RelA) specific siRNA followed by in-vitro transwell migration and invasion assays and soft agar colony formation assay. U87MG cells were harvested 48 h post-siRNA transfection and subjected to transwell migration for 24 h as well as transwell matrigel invasion for 48 h. We found 52% decrease in the migration of siRelA U87MG cells (Fig. 8a) and 30% decrease in the invasion of siRelA U87MG cells (Fig. 8b) as compared to their respective siControl treated cells.

U87MG cells were cultured and transfected with NFkB (RelA) specific siRNA. After 48 h of transfection, the cells were transferred in anchorage-independent soft agar medium and cultured for 22 days. A significant decrease in the size of the colonies formed in siRelA treated cells was found as compared to siControl treated cells (Fig. 8c). On counting the colonies of size ≥30 μm in both siControl and siRelA treated cells, we observed an average 16.3 (±2.1) colonies per field in the case of siControl cells and only 5.4 (±0.8) colonies per field in siRelA cells (Fig. 8c). *Hence, NFkB (RelA) knockdown, similar to FAT1 knockdown* [17, 19]*, inhibits cell migration, invasion and clonogenic ability in GBM cells, reflecting the importance of NFkB-FAT1 axis and their tumorigenic potential in GBM.*

## Discussion

The FAT1 gene located on chromosome 4q35.2 encodes for FAT1, a large 506 kDa transmembrane protein, belonging to cadherin superfamily [21]. Functional evidence suggests that FAT1 plays an important role in various biological processes, including developmental processes [34], cell-cell communication [20, 22], adhesion and migration [35–37]. In cancers, FAT1 is found to have oncogenic [17–19, 23–26, 31] as well as tumor suppressive role [27, 38] in context dependent manner. FAT1 expression is found to be upregulated in different cancers like pancreatic cancer [26], hepatocellular carcinoma [25], B-cell acute lymphoblastic leukemia [23], colon cancer [24] and glioma [17–19, 31] suggesting its oncogenic function in these cancers.

We have observed increased expression and oncogenic effect of FAT1 in GBM by upregulating pro-inflammatory molecules [17] as well as by promoting HIF-1α expression and signaling under hypoxia [18]. In addition, FAT1 has also been found to be essential for maintaining the clonogenic capacity of GBM cells by substantially upregulating expression of EMT/stemness markers under hypoxia [19]. The clonogenic effect of FAT1 has also been reported in U251 glioma cells [31]. The critical role of FAT1 in maintaining pro-tumorigenic microenvironment responsible for tumor progression and aggressiveness in GBM has been evident from the above reports. Furthermore, studies on FAT1 till date are mainly focused on its overexpression in tumors and its downstream functional outcome [17–19, 25, 38]. However, the upstream transcriptional regulators crucial for FAT1 expression have not been reported in literature. This study identifies and addresses the crucial role of NFкB (RelA) in the transcriptional regulation of FAT1 in GBM.

On *in-silico* promoter analysis, we observed binding motifs for various known transcription factors including NFкB-family on FAT1 promoter (− 3220 bp to + 848 bp, w.r.t. TSS + 1). NFкB (nuclear factor kappa light chain enhancer of activated B cells) is a potent transcription factor that controls transcription of a number of genes involved in inflammation, cell proliferation, invasion/metastasis etc. leading to tumor aggressiveness [9, 10, 39]. NFкB (RelA) is documented to be highly active in GBM [10, 11] and known to upregulate inflammatory microenvironment [15, 33] and tumor aggressiveness in GBM [10]. NFкB-pathway is known to be induced by tumor necrosis factor (TNFα), hypoxia, bacterial lipopolysaccharides, inflammatory stimuli etc. [15, 16, 40]. Constitutively active NFкB is seen in GBM and inhibition of NFкB-activity induces apoptosis [41]. NFкB-activity has also been associated with comparatively short survival in glioma patients [42].

We observed seventeen (17) binding motifs for transcription factor NFкB (RelA) on FAT1 promoter with high probability matrix score, suggesting a possible functional link between NFкB (RelA) and FAT1. Previous reports suggested that GBM is characterized with upregulated expression of FAT1 [17–19] and NFкB (RelA) [11, 14]. Here, observation of significant increased expression and positive correlation between FAT1 and NFкB (RelA) expression in our GBM samples (*r* = 0.70; *p* = 0.002) further strengthened this novel link between FAT1 and NFкB (RelA) expression. Tumors expressing high NFkB showed high FAT1 expression and tumors with low NFkb expression showed low FAT1. However, few samples showed high FAT1 with low NFkB expression which could be due to the regulation of FAT1 expression by transcription factors (as identified by in-silico promo software analysis) other than NFкB (RelA). However, we have not studied the clinical features of these tumors. The association of this group with any clinical phenotype needs further study in future. In addition, positive correlation between FAT1 and NFкB (RelA) expression was also observed in REMBRANDT-GBM-dataset (*r* = 0.276; *p* = 0.00004) as well as in TCGA GBM-dataset (*r* = 0.213, *p* = 0.008). The possible reason for the lower r value observed in the TCGA and Rembrandt GBM could be due to the outlier values in the bigger sample size. Similar observation of positive expression correlation between NFкB (RelA) and FAT1 in other non-glioma tumors like pancreatic, hepatocellular, stomach and lung tumors suggests the significance of NFкB (RelA)-FAT1 link in other tumors as well.

In order to confirm the transcriptional role of NFкB (RelA) on the expression of FAT1 in GBM, we have cloned and characterized 4.0 kb FAT1-promoter *pGL3F4* [− 3220 bp to + 848 bp, w.r.t. TSS (+ 1) having 17 NFкB (RelA) sites] and different FAT1 promoter-deletion constructs [*pGL3F3* (3.1 kb), *pGL3F2* (2.1 kb), *pGL3F1* (1.0 kb), *pGL3F1δ1* (0.86 kb), *pGL3F1δ2* (0.25 kb)]. Among all promoter constructs generated and analyzed, *pGL3F1* [1 kb; − 200 bp to + 848 bp, w.r.t. TSS(+ 1) with 3-NFкB (RelA) binding sites (at -90 bp/− 80 bp, + 347 bp/+ 356 bp and + 360 bp/+ 368 bp) showed the highest promoter activity in GBM cells (U87MG/A172/U373MG) reflecting the potentially strongest site for NFкB (RelA) activity. The promoter (pGL3F1) deletion analysis indicated NFкB (RelA) site at -90 bp/− 80 bp on FAT1 promoter to be critical for NFкB (RelA) activity.

And since hypoxia and TNFα are known activators of NFкB pathways in GBM [30, 33], on exposure of *pGL3F1*-FAT1-promoter transfected GBM cells to severe hypoxia (0.2% O_2_), TNFα and NFкB (RelA) (RelA+IKBK overexpressing vectors) increased the *pGL3F1*-promoter activity. Significantly increased levels of endogenous-FAT1 transcripts and FAT1 protein level were also observed. Furthermore, siRNA-knockdown of NFкB (RelA) significantly downregulated the FAT1 mRNA-expression, FAT1 promoter activity as well as reduction in FAT1 protein level. Moreover, 5′ deletion of NFкB (RelA) motif at -90 bp/− 80 bp from pGL3F1 as well as site directed mutagenesis (pGL3F1mtRelA) of NFкB (RelA) motif at -90 bp/− 80 bp from pGL3F1 resulted in significant reduction in the promoter activity. In addition, the endogenous binding of NFкB (RelA) at -90 bp/− 80 bp on FAT1 promoter was also confirmed by ChIP assay. These findings validated and confirmed NFкB (RelA) as a potential transcription factor regulating FAT1 expression in GBM.

Our findings signify the regulatory importance of NFкB (RelA) on FAT1 mRNA expression. NFкB is one of the master regulators of pro-inflammatory genes in cancer which upregulates inflammatory-microenvironment in cancer by increasing the expression of cytokines and chemokines [15, 16] as well as in regulating cell proliferation, apoptosis, cell-morphogenesis, cell-differentiation etc. in cell/context dependent manner [9–13, 16]. Hypoxia has long been identified to co-exist with chronic inflammation in tumors [15, 16, 28, 29] and HIF-1α, a master regulator of tumor hypoxia, is known to be regulated in NFкB dependent manner and cooperatively maintain malignant phenotypes in cancer [16, 43, 44]. We had initially demonstrated that FAT1 is a major contributor to pro-tumorigenic inflammation in gliomas driving the upregulation of pro-inflammatory cytokines like IL6 and IL1ß and also COX2 via AP1 activation [17]. Subsequently, we have also shown FAT1 to up-regulate HIF1α at the level of both transcription and translation via the AKT/mTOR pathway [18], and simultaneously increasing cellular invasion. We have also demonstrated FAT1 to affect EMT and stemness, both independently and also via HIF1α in GBM under hypoxia [19]. Interestingly, activated NFкB also contributes to epithelial-mesenchymal transition and promote invasion/metastasis [9]. Tumor cells with activated NFкB contribute to maintenance of stemness in different tumors [16, 45–47]. On knocking down NFкB (RelA) in GBM cells, a significant decrease in the migration, invasion and colony forming capacity of U87MG cells was observed. Interesting parallels between the activity of NFкB and FAT1 have been demonstrated, at least in glioma. However, as NFкB (RelA) is known to affect several distinct downstream pathways, the exact contribution of the NFкB-FAT1 axis cannot be determined by this study and is beyond the scope of this work. Future studies are needed to validate the extent of this axis. The positive correlation of FAT1 and NFкB (RelA) in other tumors like pancreatic, hepatocellular, lung and stomach cancers suggests that the same mechanism may be operative in these tumors also. However, this is yet to be confirmed experimentally. The role of NFкB regulated pathways in maintaining and promoting hypoxia, inflammation and stemness in cancer cells has been an area of significant scientific interest and many NFкB/HIF-1α inhibitors are under clinical cancer trials [48–51]. Our results regarding the role of NFкB (RelA) in upregulating FAT1 transcription and in regulating cell migration/invasion and clonogenecity support the possibility that the pro-tumorigenic responses by NFкB (RelA) may be partly mediated through the NFkB-FAT1 axis.

## Conclusion

This study shows a novel link between NFкB (RelA) and FAT1 at the molecular level. Hence, we suggest that the NFкB-FAT1 axis may have clinical relevance as a potential therapeutic target for combating protumorigenic inflammation, hypoxic response, stemness and EMT related phenotypes. While this study has been conducted in GBM, this axis may be relevant in other tumors as well.

## Supplementary information

**Additional file 1: Figure S1.** BLAST alignment of FAT1 gDNA sequence and FAT1 transcript sequence. **Figure S2.** In-silico analysis of FAT1 promoter sequence showing binding motifs for potential transcription factors. **Figure S3.** In-silico analysis of FAT1 promoter sequence showing **(a)** partial sequence of the FAT1 promoter region -3220 bp to +848 bp w.r.t. TSS (+1) with binding motifs for potential transcription factors and **(b)** NFкB (RelA)/RelA binding sites and dissimilarity rate (matrix score) on FAT1 promoter (4.0kb). **Figure S4.** Correlation of NFкB (RelA) and FAT1 expression in different tumors from Rembrandt database. **Figure S5.** Output of nucleotide BLAST search, performed using 4.0kb putative FAT1 upstream region. **Figure S6.** In-silico analysis of core promoter region of FAT1 gene. **Figure S7.** Luciferase activity of 5’ deletion constructs of FAT1 promoter constructs (pGL3F2): U87MG cells were transfected with pGL3F2 (2.1 Kb) and its 5’Deletion constructs [pGL3F2δ1 (1.3kb) and pGL3F2δ2 (1.1kb)] along with pGL3F1 for 48 hours. **Figure S8. a)** Gradient PCR (annealing temperature range-50°C to 70.5°C) was performed to confirm the annealing temperature of NFkB (RelA) SDM primers. PCR amplified product of mutated construct (pGL3F1mtRelA) was observed at the annealing temperature at 69.7°C (Lane 3), 68.1°C (Lane 4) and 66°C (Lane 5) by gel electrophoresis analysis. **b)** pGL3F1 [wild type NFkB (RelA) site] was taken as parental plasmid to amplify muatated consruct (pGL3F1mtRelA), further parental plasmid was digested with DpnI restriction enzyme followed by gel electrophoresis analysis. **Figure S9.** Confirmation of NFkB (RelA) mutation in pGL3F1mtRelA construct by EcoRI digestion and sequencing analysis. **Table S1.** List of PCR Primers. **Table S2.** List of various FAT1 promoter constructs. **Table S3.** Nucleotide sequence of PCR primers used for qPCR, ChIP assay and site directed mutagenesis.

## Data Availability

All data generated or analysed during this study and materials used are available in the manuscript and supplementary information file.

## Abbreviations

ChIP: Chromatin-immunoprecipitation
EMT: Epithelial-Mesenchymal Transition
GBM: Glioblastoma
HIF-1α: Hypoxia Inducing Factor- 1α
NFкB: Nuclear Factor κ B
TNFα: Tumor Necrosis factor α
TSS: Transcription Start Site
