# Supplementary material for: NFкB is a critical transcriptional regulator of atypical cadherin FAT1 in glioma
Source: BMC Cancer. 2020 Jan 28;20:62. doi: 10.1186/s12885-019-6435-1 (PMC6988320; doi:10.1186/s12885-019-6435-1)
Supplement: Supplementary file 1 — Additional file 1: Figure S1. BLAST alignment of FAT1 gDNA sequence and FAT1 transcript sequence. Figure S2. In-silico analysis of FAT1 promoter sequence showing binding motifs for potential transcription factors. Figure S3. In-silico analysis of FAT1 promoter sequence showing (a) partial sequence of the FAT1 promoter region -3220 bp to +848 bp w.r.t. TSS (+1) with binding motifs for potential transcription factors and (b) NFкB (RelA)/RelA binding sites and dissimilarity rate (matrix score) on FAT1 promoter (4.0kb). Figure S4. Correlation of NFкB (RelA) and FAT1 expression in different tumors from Rembrandt database. Figure S5. Output of nucleotide BLAST search, performed using 4.0kb putative FAT1 upstream region. Figure S6. In-silico analysis of core promoter region of FAT1 gene. Figure S7. Luciferase activity of 5’ deletion constructs of FAT1 promoter constructs (pGL3F2): U87MG cells were transfected with pGL3F2 (2.1 Kb) and its 5’Deletion constructs [pGL3F2δ1 (1.3kb) and pGL3F2δ2 (1.1kb)] along with pGL3F1 for 48 hours. Figure S8. a) Gradient PCR (annealing temperature range-50°C to 70.5°C) was performed to confirm the annealing temperature of NFkB (RelA) SDM primers. PCR amplified product of mutated construct (pGL3F1mtRelA) was observed at the annealing temperature at 69.7°C (Lane 3), 68.1°C (Lane 4) and 66°C (Lane 5) by gel electrophoresis analysis. b) pGL3F1 [wild type NFkB (RelA) site] was taken as parental plasmid to amplify muatated consruct (pGL3F1mtRelA), further parental plasmid was digested with DpnI restriction enzyme followed by gel electrophoresis analysis. Figure S9. Confirmation of NFkB (RelA) mutation in pGL3F1mtRelA construct by EcoRI digestion and sequencing analysis. Table S1. List of PCR Primers. Table S2. List of various FAT1 promoter constructs. Table S3. Nucleotide sequence of PCR primers used for qPCR, ChIP assay and site directed mutagenesis. [file 12885_2019_6435_MOESM1_ESM.docx]

**Supplementary figures:**

**Figure S1**

**Figure S1: BLAST alignment of FAT1 gDNA sequence and FAT1 transcript sequence:** Nucleotide BLAST was done between FAT1 transcript sequence (+1bp to +193bp) reported by Ensembl genome browser and human gDNA. The BLAST analysis showed 100% complementary between FAT1 gDNA sequence (+1bp to +193bp) (black color) to FAT1 transcript sequence (brown color) (+1bp to +193bp). 5’ upstream sequence of FAT1 gDNA from transcription start site (+1) represents proximal FAT1 promoter region. The nucleotide has been numbered with reference to transcription initiation site, which is assigned +1 nucleotide number.

**Figure S2**

CATCGCGACG AATTTCGGCT GCAGAAGAGG AAGAGCATCA CCTGCAGTCC -3190

NFkB(RelA)/NFkB1/NFkB

NFkB(RelA)/NFkB

c-myc

**FAT1 Forward primer**

GCGGCGTGGG AAACGCCGCC GGAGGGGCTC CGCGAGCCGG GGAAGTGCGC GCAGCGGGGC -3130

TCCAGGCGGC TCCAGGCGCT GAGTGCGAGG AACGCCCGCG CTCGCCGGCG CCCGGGAGAC -3070

GCAGCCGAAG GAGGAGGGCG GGCGTTTCAC CCGCGCCACA GCTGCGGCCG GCCAGAGAGA -3010

AP2beta

AP2beta

CCGCAGAGGC CGGGACCCCC GCAGCCTCCA CGGCCGCTGC TGCCACTGTC GGGGTGGCGC -2950

c-jun

GCCCAGCCGG AGAATCCCGT CCGCGCCCGG GGCGGGGGGC GAGGCGGGGC GCTGACGTCA -2890

CCTCCCCTCC CCCACCGCCT CCAAGGGGGA GGGAGTGGGG AAAGAGGCAG CGAGTCGCCG -2830

c-jun

CAGCCGGCGG CCGCAACTCC TCAACTCGGC GGCTGGCTGC CTGGGCACGT CCTGACAAAC -2770

TTGGCTGCTC CCGGGCCCTC GTTCCGGCCC CGCGCACTCT CTTTTCGCCT GCGTTTGGTG -2710

CTTAAGTTTG CCCTGGTCGG AAGCCTCCGG AGATGCGGCC GCCTGGGTCC CGGCGGCACG -2650

AP2beta

GACGTTGGCA CTCTGGTTAG GGACATGCTG AAGATCCCGG GGAGGCAAAG TTGGTACTTC -2590

AAACCCGGCT CTTAACACAT ATCTACACCG CGAGGGGATC ACGGAGCTGC CGAGGACTTT -2530

AGAGCCCAGG GCCGAGGACT CGGCTAGACT GGTGAGTTCT TTGTTCAAAT CGTGCATGCC -2470

TTTTGGCATT TAAGTTTAAA CGTTAATGAA ATTACGTGGG GTGTGTGGCT CTAAGCGAGT -2410

NFkB(RelA)

GCGGGGAAGC GTGTGCGCCC TACGGGTTCT CGGCTCCAGG AGCTGCGCAT CCCTCTGGCT -2350

TTGTGCGGAT CGGCGATGAA AGATTCGTAT GCATAGCTTT GGGAGTTCAT AAAATACATA -2290

TAGAGCACTG TTAAAAAGAA CTTGAGAGGC GATGCTGTTT GTGCATTATT TTGAATGTAA -2230

CTTATTATAG CAATGGTGCC TAGGGAGGGC GCATCGGGGC CGCGAGATTT GCAGGGGCGC -2170

c-myc

AGCGCCCTCT AGTCCGGGCA CCTGGCGAGG CGCGCTCCTT GCAGGGTTCT GTGCGGAGGC -2110

NFkB1

c-jun

GAACCTGGAC CTCCCCGGCG CTCGGCGACG TGGGCGCTTG CTCACCCCAG TCTTCACTGA -2050

CCACCCACCC GGGCCCATTC TTTATATGAG TTACAAAAAT CAGCTTGGGT GGACTGGATG -1990

AP2beta

TAGAGGCCGT GCTGGAGGAA AGCGTTTTGT TTCTGGAACC GTCTCCCCAA CCCCCTCCCT -1930

AATTTCCACG AGCCTTTGGG GACAGGGTTA GGGAGGGTCG ATTATTTAAA GGACAAATGT -1870

GGAACTTGAA CCACATTTCT CTCTCTCATA TAAAATAATT CGATTTGTGG TAGCTAATAG -1810

GCATGTCCAT TTCGTTCTGT TGGCGCTTAA GGATCAGTAG AATCGTGGCA GAGTCCTAAG -1750

TCTGAAATCT CCATCCCTGG CTCTCGGCGG CTCCTGTGGC GACGAAGTGC AACTTCTTCC -1690

NFkB(RelA)/NFkB

AP2beta

AGGCAGATAG ATTTCTCAGG GAGCCTGGAA AATCGGGATT CCCAGACAGA TGTAAAACAT -1630

CTAGTAAGCC CTAGGAAGCG TTATTACTTA TTAATTAATG GTAATCTTCT CCAGGAATTC -1570

NFkB(RelA)

c-jun

TCTTGTGCCA TTTAAAAAAA ATCCCGTGAT TTTACGTCAG GGAATGGTAT CTTTGTACTT -1510

NFkB(RelA)

TTTTTACTGT TCTTCTTTTC CCCTTTTAGC ATTACTCCCG CTTCCTCGCT CCCAGGAAAC -1450

GGCTGGAGTT CCGGTGTGTT TGGGTTTTGC TGAGATTTAC AATTCGGCAA GCGGCACAAG -1390

c-jun

CCGGTTGGGC TTGGAGATCA GGTCACCCAC TTTCGAGGTG TTCCATGTAT GAAATTGACC -1330

c-myc

TGTAAGCACT TGGAGCAATT ATGGCCGGGA CAAACACGTC TTTGGATGAT GGAGTTTGCA -1270

NFkB(RelA)

TTTGCTGGAT GTGTGAATCT ATGTGTATTC CAACTGGTGG GCAGGGAATA TAGGGCTACG -1210

AATCGGCACA CTCATCTTGC AGGCATTATT TTTCCTTCCC AGCCACAACC TGCTAGACAC -1150

AP-1

c-jun

CTTTATTATT ATTATTTTTT CCGGTCACCT CTAAGATGGC TTATAAGGTG ACTGCGTTAA -1060

NFkB(RelA)/NFkB

/c-jun/AP1

c-myc

CTTTGAACCC AGGAAATTCT CTTTCACACT TGACTGCCCT GCCGTTCCCC TCCCCGCCAA -1030

NFkB(RelA)/NFkB

GCTCCCCTCA CCAGTCCCAG GGAAATCGGA TCTCTTTGTC TTCCCAGTGT TAAAATGCCT -970

GAGTCCCACA AACTTTCCGA GATCAAGTCC GCCCTCTTGT CCGGGCTGCG TCCCAGCGCC -910

TCCGGCCCTC CGGGCTTCGG CCGAGTCCGC CCTGCGCCGA GCCTGCGGCT GGAGCCCTGC -850

CCGCACCGGG GATGGGCTCC GCGCCCGGGA CGCTCAGGCT GGGCGCCCGG CGCGGGGAGT -790

NFkB(RelA)/NFkB1/NFkB

TCTCGGAGCG GGCTTCTCGG CGCTGGGGCT TCCCCTCTGC CTAGGAACTT GCAGTTGTCG -730

c-jun

AP-1 beta

GCCTGGGGTC ACGCTTTGGG GCCCCTGGGG CCGACTCCCG GGCTAGGAAG CCCGCGGGAC -670

c-jun

GCCGCGACTG CCTTTTGTCC CACAGGCCTG GGCGTCCGGG GTGTCAGCGG GGGAGGCCAG -610

AP-1 beta/NFkB1

NFkB(RelA)

GCGCCCGCGG CGGGGAGGGC CTGGGGTCTA ACTCGGCAGA AAAAGGTCGC GGGGGAAGCG -550

TTTCCATTTT CTGGGGATTG AAGGAGGAAG TAGATTCCGA CCTAGCTGCA TTCCTTTGGA -490

GACCTGAAGG ACGACTTCAG CCCACCAAAC CTTCGCGGAC CCAGAGCCGC GCCATTCAAA -430

NFkB(RelA)

TGCAGATTCA GGGGCTCCCA GAAGGCTATG GAAAAGCTAA AGGGATGCCT CTGGGAAGCT -370

NFkB(RelA)

TTCTCATTAG GCCCCAGCCT TTTTTTTTTT TTTTTTTTTT TAGCTAAGCT AACGTTTCCC -310

NFkB(RelA)/NFkB

TTTCTTGGCA GGGAGCTTTC CCCAATTCCT TGCACAGCGC CTGTGGCTGG CCGTAACTAA -250

CCTCGGCATC ACAAGCCTCG CCCTGCCCCT CGGAGCCGCG CTAGACCCCG CTGGGCGCCT -190

TCTCGTCTCC CAGCACGGAC CTGGTGTTCG CAGGGACTGG GGCTCTTTGG GTCCGGGCGG -130

NFkB(RelA)

GACTGCGTGG CCGAGCCCGC GGTCCCAGAG CCGGCAGCCG GGGAAAGTGG GCTCGAGTTT -70

CCGCGCCCGA GAAATGAGCG CCGGCGCCGG CGGGCGGGGC CGGGGCGGAG GGATCTCCTG -10

AP-1 beta

**+1**

**CGCTCGGGGA GCGTGAGGCG** CCGGCGCCGA GCTGGGCGGC CGGGCGCGGG GAGAGGGCGC +51

GGGAGCGGCT CGTGCGGCAG GTACCATGCG GACGCGCGAG CCCGGCGAGG GCCCCGGCAG +111

GCCCGGTCCC TGCTCGGGGG CGCGCTGAGA CGGCGGGTGA GCTCCACGAG AGCGCCGTCG +171

AP-1 beta

CCACTTCGGG CCAACTTTGC GGTTAGTTCG CTGGGGCGCG GGCCGGCGGG CGGGGGCGAG +231

AGCGTGCAGC GCGCTGCGGT GCGGACTCTG TGGGTGTCCG CCCGGTCCCC GGCTCGGGCT +291

NFkB(RelA)

c-jun

CCGGCGCTGC CGAAAACGGA TGAGCGCGCG ACTTCCCGGA GGGTGGAGTG ACTCGGTCCT +351

NFkB(RelA)

AP-1 beta

TCCCAGTTGG GAAATTCAGC ACTGGCTAGG GGGGTCCCGG CGGCGGAGCG GGAAAAGGAG +411

c-jun

GCGACCGCGC GGGACCGATC CGGGGCCATT GCTGGGGTCC GATCCTTTCT GACACGCCGC +471

CTTCCCTCCG GGAGCTCGCC GGGCTGCGCC GGCCTCGCGG TCTCCTGGGG CATCCCGCCG +531

AP-1 beta

TCTGCGAAAG ACCCGCTCGC TGAGCGGTGC ACGCGGGAGG CCTGCGCGGG GCTGCCGGGT +591

CCCTGGGTCT CTGGGTGTTT GCGCCCTGCA GGAGCGCTGC CCGGAGGTGC AGATGCAACC +651

GGTGGACTGC GGAGTGAAGG GCTGCGAGGC TGCATTTCGG GGCTGAACAT CTGCACAATG +711

c-jun

TGCGGTTTGT CAGTGTTTTC TGTCTTCTCT GGAGGAGGAA CTTCTAAGAT CTTTTTAAAA +771

GAAAGAGTTT TTGGTCCTTC ATTGCCTTTA AAAAAAAATG CTTTCTTTTA GAGAGCCAGC +831

TTTCTGCTGT GTGTTAG +848

**FAT1 Reverse Primer**

**Figure S2: In-silico analysis of FAT1 promoter sequence showing binding motifs for potential transcription factors.** The figure shows sequence of the FAT1 promoter region -3220 bp to +848 bp w.r.t. TSS (+1). Transcription factor (TF) binding motif analysis was done by Promo Alggen (http://alggen.lsi.upc.es/) program using TRANSFAC 8.3 database (matrix dissimilarity upto 15%). NFkB(RelA) and other TF(s) binding motifs identified are marked. There are 17 NFkB(RelA) binding motifs in the 4.0kb FAT1 promoter region. The transcription start site (TSS) is marked as +1. FAT1 specific primers (forward and reverse) used to amplify FAT1 promoter region (4.0kb) is shown in yellow box with arrow.

**Figure S3**

**(a)**


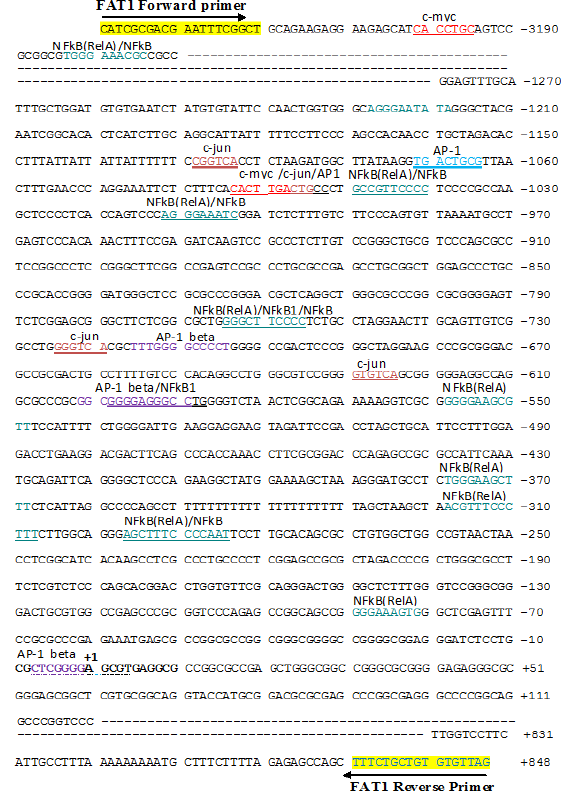


**(b)**

**
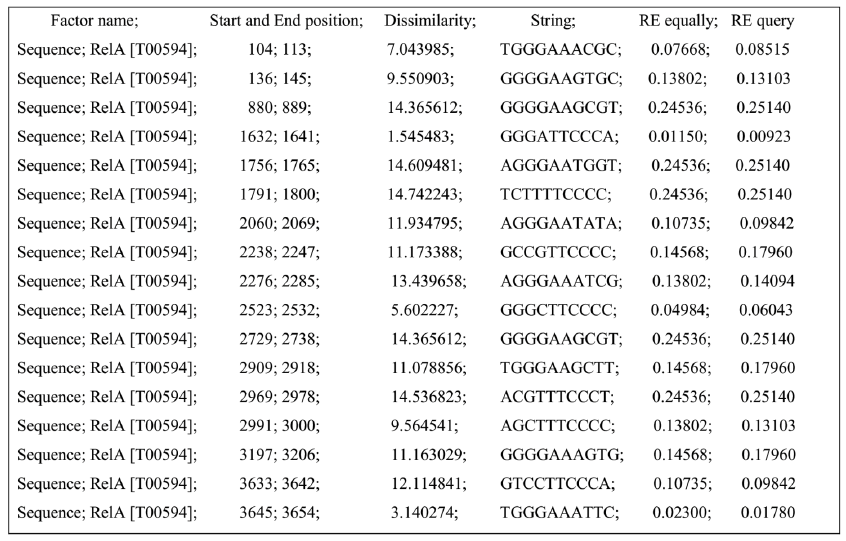
**

**Figure S3:** In-silico analysis of FAT1 promoter sequence showing **(a)** partial sequence of the FAT1 promoter region -3220 bp to +848 bp w.r.t. TSS (+1) with binding motifs for potential transcription factors and **(b)** NFкB(RelA)/RelA binding sites and dissimilarity rate (matrix score) on FAT1 promoter (4.0kb). Transcription factor (TF) binding motifs analysis was done by Promo Alggen (http://alggen.lsi.upc.es/) program using TRANSFAC 8.3 database (matrix dissimilarity upto 15%). There are seventeen (17)NFкB(RelA) binding motifs in 4.0kb FAT1 promoter region. The transcription start site (TSS) is marked as +1.

**Figure S4**

**(a)**

**
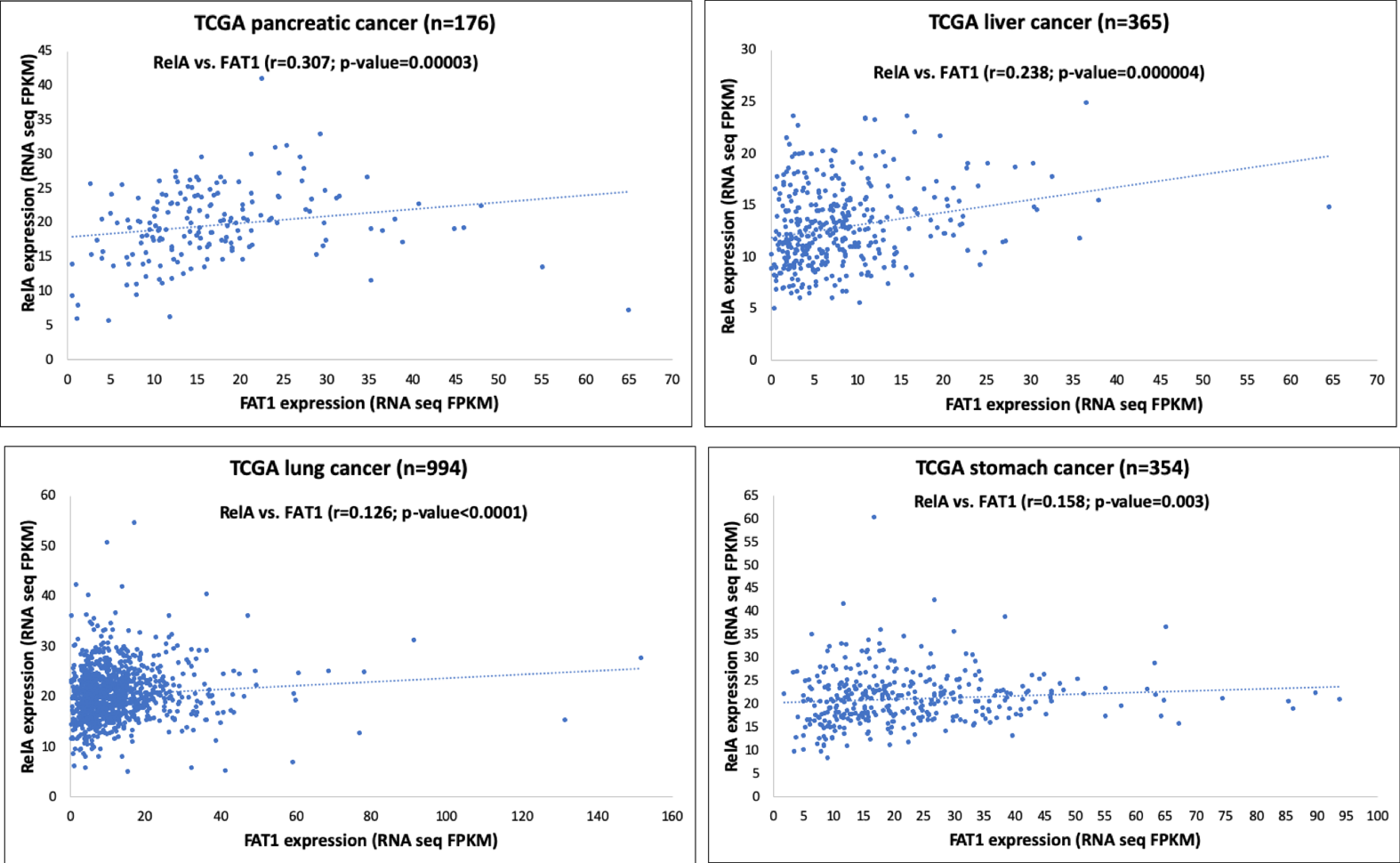
**

**(b)**

**(d)**

**(c)**

**Figure S4: Correlation of NF**к**B(RelA) and FAT1 expression in different tumors from Rembrandt database.** Expression values of NFкB(RelA) and FAT1 were obtained from Rembrandt database. Significant positive correlation was found between expression of NFkB(RelA) and FAT1 in **(a)** pancreatic cancer cases (n=176), (r=0.307, p=0.00003); **(b)** liver cancer cases (n=365), (r=0.238, p=0.000004); **(c)** lung cancer cases (n=994), (r=0.126, p<0.0001); and **(d)** stomach cancer cases (n=354), (r=0.158, p=0.003)

**Figure S5**


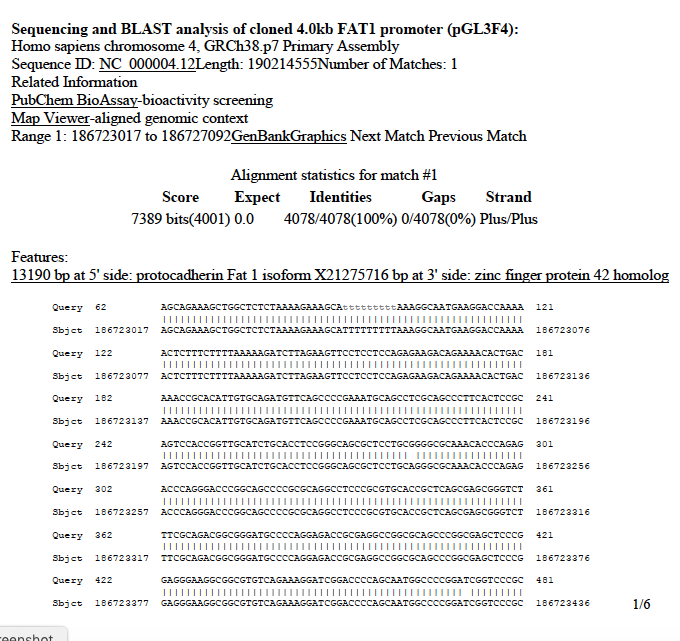


**
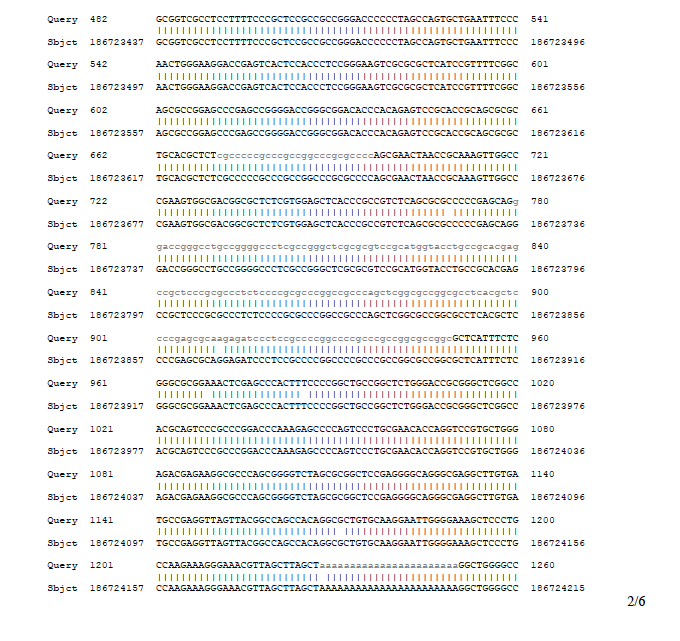
**

**
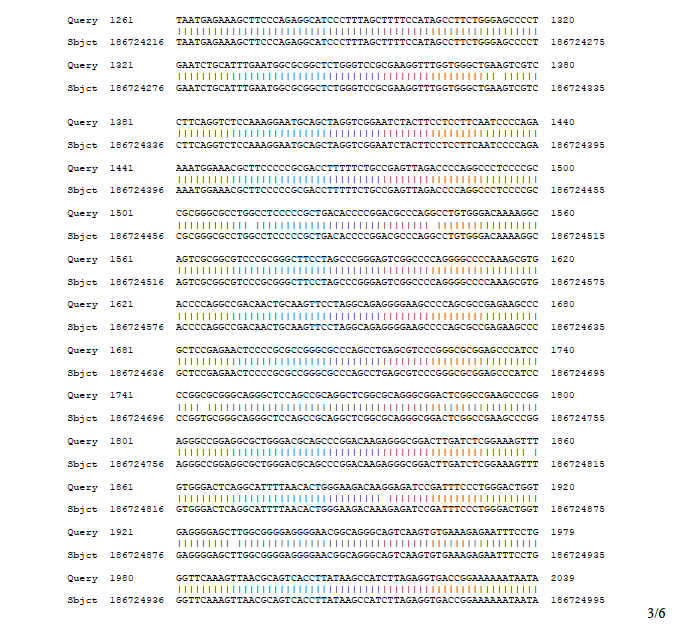
**

**
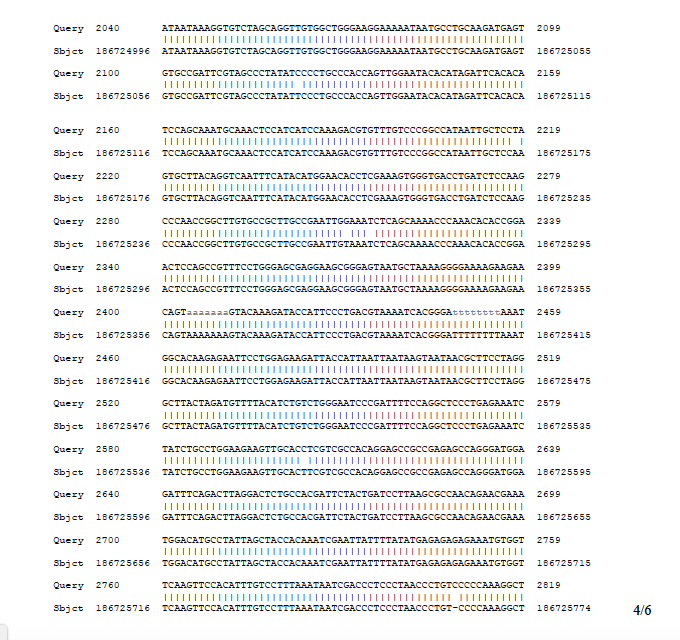
**

**
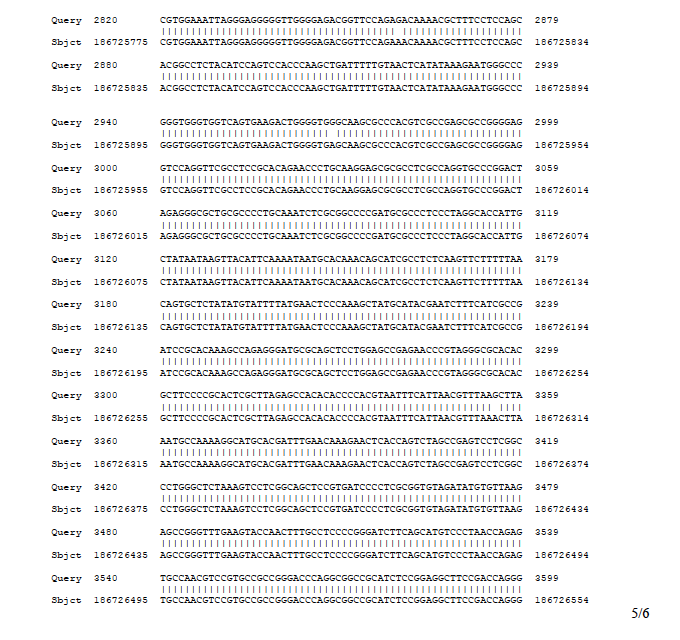
**

**
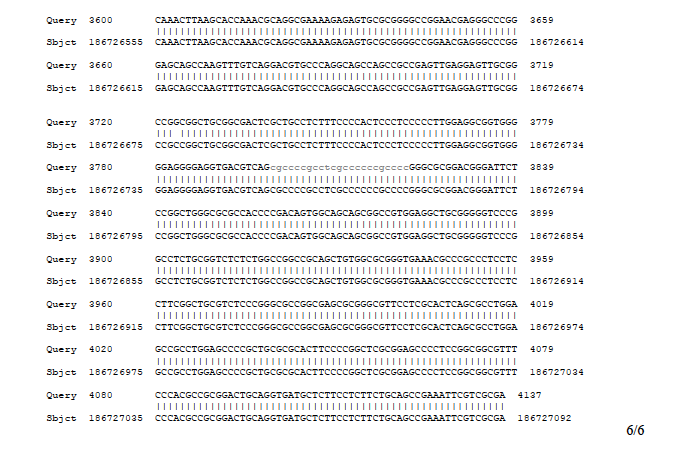
**

**Figure S5: Output of nucleotide BLAST search, performed using 4.0kb putative FAT1 upstream region.** A nucleotide blast search was performed using cloned 4.0kb FAT1 fragment. The result showed complete homology of this sequence with a region (186723017bp-186727092bp) of chromosome - 4 present upstream to the sequence coding for FAT1 gene. The sequence of PCR amplified fragment is shown as query sequence.

**Figure S6**

**Figure S6: In-silico analysis of core promoter region of FAT1 gene: a)** Nucleotide sequence of core promoter region of the FAT1 gene -238bp/+193bp w.r.t. TSS. **b)** TATA box, CAAT box, CG was analysed by Eukaryotic promoter database (EDP software). FAT1 promoter (-238bp/+193bp) sequence was subjected to EPD software. Transcription start site (TSS) is marked as+1. FAT1 gene lacks TATA box (black box) and CAAT box (red box) and rich in multiple GC box (green box).

**Figure S7**


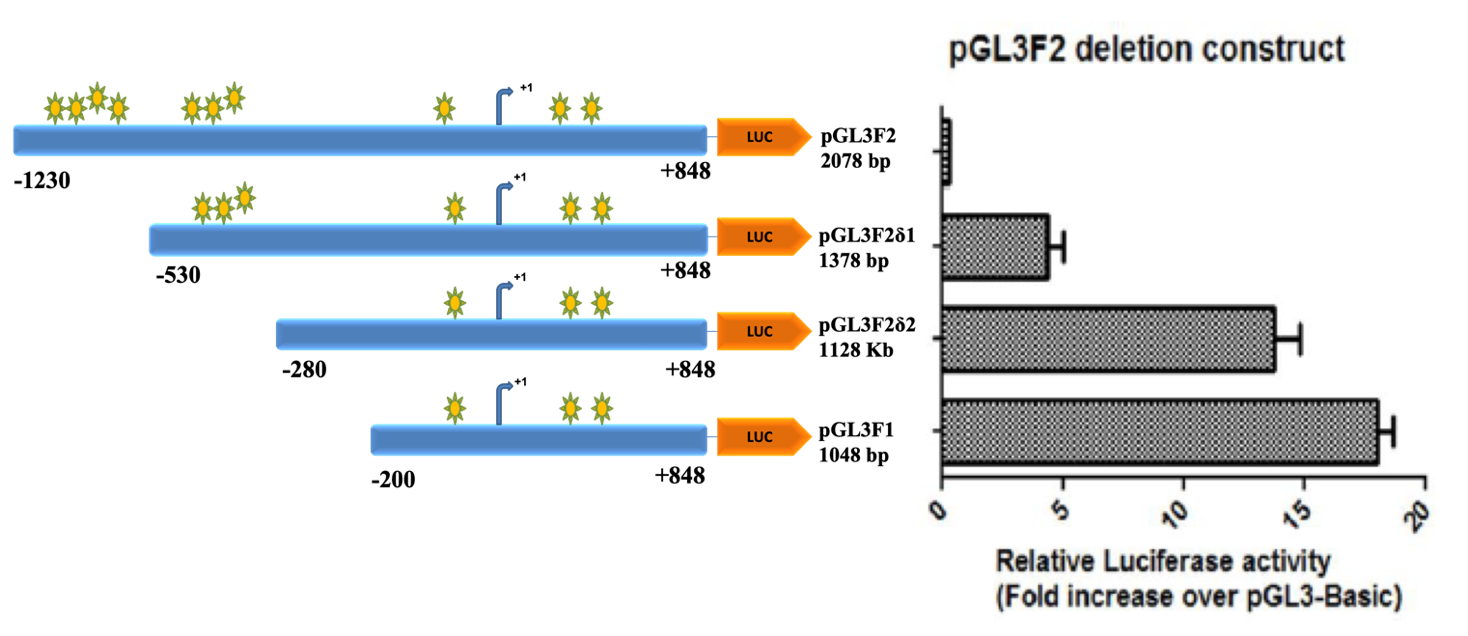


**Figure S7:** Luciferase activity of 5’ deletion constructs of FAT1 promoter constructs (pGL3F2): U87MG cells were transfected with pGL3F2 (2.1 Kb) and its 5’Deletion constructs [pGL3F2δ1 (1.3kb) and pGL3F2δ2 (1.1kb)] along with pGL3F1 for 48 hours. pGL3F1 showed highest luciferase activity (18.02 fold ±0.6) followed by pGL3F2δ2 (13.7 fold ±1.08), pGL3F2δ1 (1.81 fold ±0.50) and pGL3F2 (0.27 fold±0.01) as compared to pGL3B. Bar graph represents fold luciferase activity of FAT1 promoter constructs (Normalized to pGL3Basic and internal control pRLTK). Values are the mean ± SE of at least three independent experiments performed in triplicate.

**Figure S8**


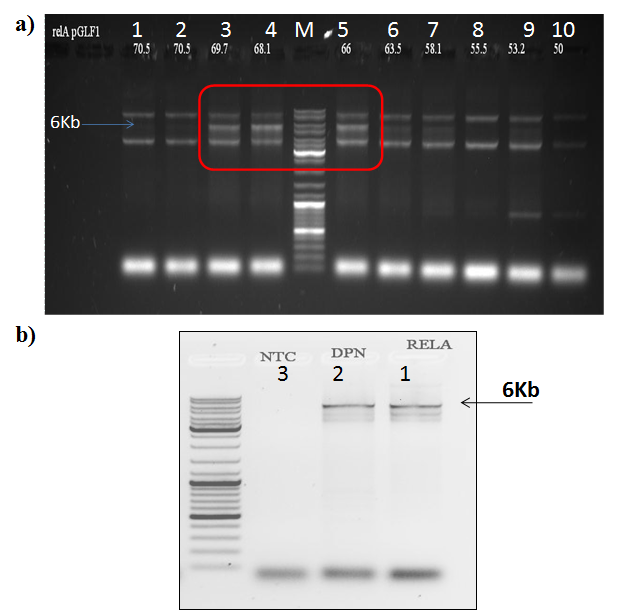


**Figure S8: a)** Gradient PCR (annealing temperature range-50°C to 70.5°C) was performed to confirm the annealing temperature of NFkB(RelA) SDM primers. PCR amplified product of mutated construct (pGL3F1mtRelA) was observed at the annealing temperature at 69.7°C (Lane 3), 68.1°C (Lane 4) and 66°C (Lane 5) by gel electrophoresis analysis. **b)** pGL3F1 [wild type NFkB(RelA) site] was taken as parental plasmid to amplify muatated consruct (pGL3F1mtRelA), further parental plasmid was digested with DpnI restriction enzyme followed by gel electrophoresis analysis. Lane 1: PCR amplified product of NFkB(RelA) mutated construct (pGL3F1mtRelA)-product size-6kb Lane 2: DpnI digestion: NFkB(RelA) mutated construct (pGL3F1mtRelA)-product size-6kb, Lane 3: Negative control

**Figure S9**

**
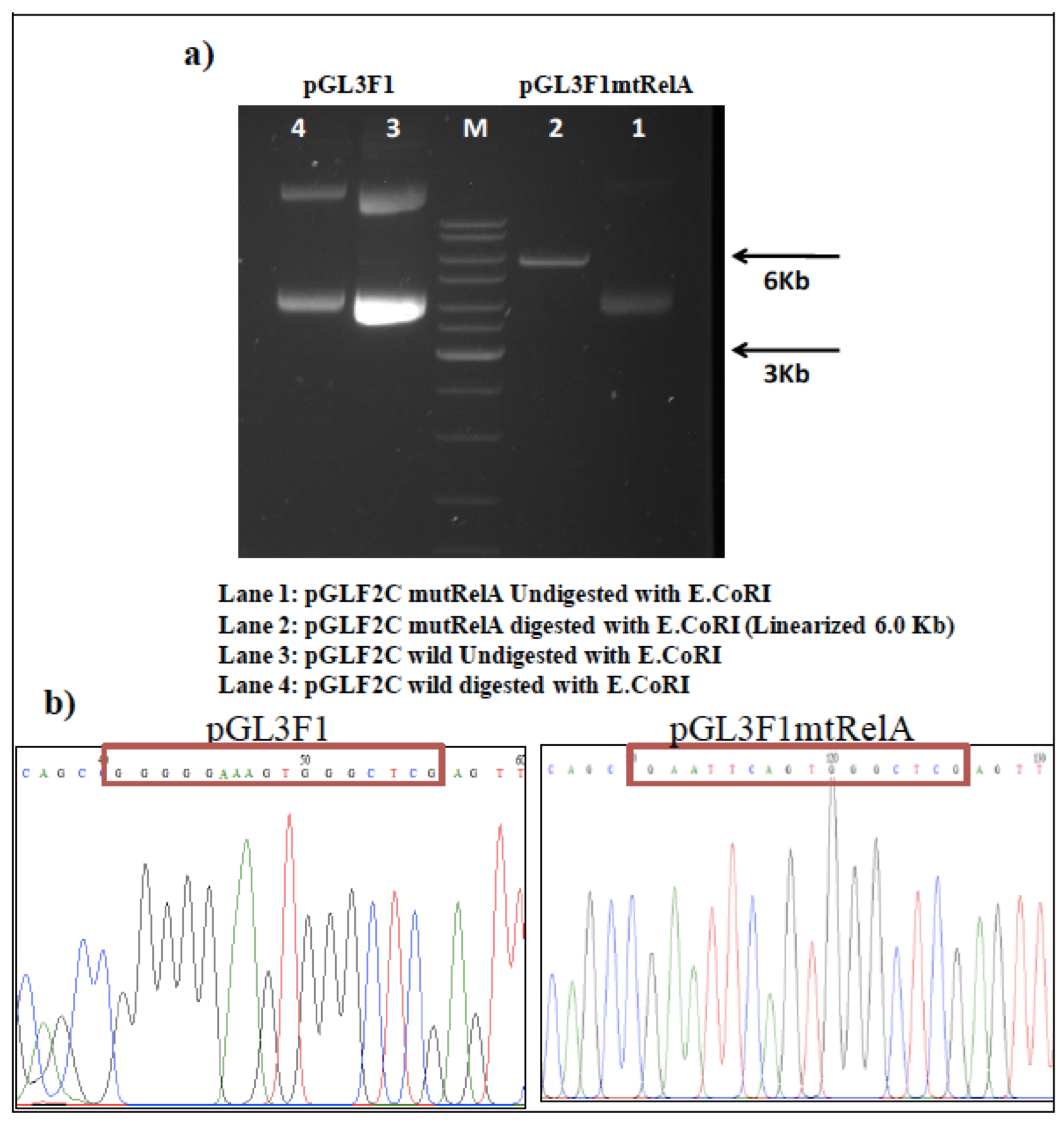
**

**Figure S9: Confirmation of NFkB(RelA) mutation in pGL3F1mtRelA construct by EcoRI digestion and sequencing analysis: a)** pGL3F1 was taken as parental plasmid and primers were designed with mutated RelA site to amplify by PCR followed by DpnI digestion and transformation. Plasmid was confirmed by EcoRI digestion. Wild type vector (pGL3F1) and mutated vector (pGL3F1mtRelA) were subjected to EcoRI digestion at 37°C and analyzed by gel electrophoresis. Wild type vector (pGL3F1) could not get digested and mutated vector was digested and released linearized 6.0kb product. Lane 1: Digested pGL3F1, Lane 2: Undigested pGL3F1, Lane 3: Digested mutated vector (pGL3F1mtRelA) (6.0 kb) and lane 4: Undigested mutated vector (pGL3F1mtRelA). **b)** Confirmation of mutated NFkB(RelA) sequence by chromatogram analysis of wild type (pGL3F1) and NFkB(RelA) mutated vector (pGL3F1RelA).

**Supplementary tables:**

**Table S1:** List of PCR Primers: **Nucleotide sequence of PCR primers used for amplification of upstream region of FAT1 gene.** The PCR primers were designed by Primer 3 software, based upon the nucleotide sequence of FAT1gene given in human genomic data base (Accession no. **NC_000004.12). Forward primers were flanked with MluI restriction site (5’**ACGCGT3’**) and reverse primers were flanked with NheI restriction site (5’**GCTAGC3’**).** The nucleotides have been numbered with reference to transcription initiation site, which was assigned +1 nucleotide number.

| FAT1 Promoter constructs | FAT1 promoter (insert) | Sense (5'to3') | Position (bp) wrt TSS (+1) | AntiSense (5'to3') | Position (bp) wrt TSS (+1) | Product size (kb) |
| --- | --- | --- | --- | --- | --- | --- |
| pGL3F4 | F4 | GGCGACGCGTCGCGACGAATT | -3220 | AACAGCTAGCAGAAAGCTGGCTC | +848 | 4068 |
| pGL3F3 | F3 | CTTGAGACGCGTTGCTGTTTGTGC | -2260 | AACAGCTAGCAGAAAGCTGGCTC | +848 | 3108 |
| pGL3F2 | F2 | TTCCGGTCACGCGTAAGATGGC | -1230 | AACAGCTAGCAGAAAGCTGGCTC | +848 | 2078 |
| pGL3F2δ1 | F2δ1 | AAGGACGCGTTAGATTCCGA | -530 | AACAGCTAGCAGAAAGCTGGCTC | +848 | 1378 |
| pGL3F2δ2 | F2δ2   \| F2δ2 \| \| --- \| | TGCACACGCGTTGTGGCTGG | -280 | AACAGCTAGCAGAAAGCTGGCTC | +848 | 1128 |
| pGL3F1 | F1 | AGGGACGCGTGGCTCTTTGG | -200 | AACAGCTAGCAGAAAGCTGGCTC | +848 | 1048 |
| pGL3F1δ1 | F1δ1 | CGGACGCGTCTCCTGCG | -20 | AACAGCTAGCAGAAAGCTGGCTC | +848 | 868 |
| pGL3F1δ2 | F1δ2 | AGGGACGCGTGGCTCTTTGG | -200 | CGCTAGCGCGCCCTCTC | +50 | 250 |

**Table S2: List of various FAT1 promoter constructs:** The nucleotides have been numbered with reference to transcription initiation site, which was assigned +1 nucleotide number.

| **Construct name** | **Insert name** | **Start 5’** | **End 3’** | **Size of insert (bp)** | **No. of RelA sites present** | **No. of RelA sites removed** |
| --- | --- | --- | --- | --- | --- | --- |
| **pGL3F4** | F4 | -3220 | +848 | 4068 | 17 | 0 |
| **pGL3F3** | F3 | -2260 | +848 | 3108 | 13 | 4 |
| **pGL3F2** | F2 | -1230 | +848 | 2078 | 9 | 7 |
| **pGL3F2δ1** | F2δ1 | -530 | +848 | 1378 | 6 | 11 |
| **pGL3F2δ2** | F2δ2 |    \| -280 \| \| --- \| | +848 | 1128 | 3 | 14 |
| **pGL3F1** | F1 | -200 | +848 | 1048 | 3 | 14 |
| **pGL3F1δ1** | F1δ1 | -20 |    \| +848 \| \| --- \| | 868 | 2 | 15 |
| **pGL3F1δ2** | F1δ2 | -200 | +50 | 250 | 1 | 16 |

**Table S3: Nucleotide sequence of PCR primers used for qPCR, ChIP assay and site directed mutagenesis**

| **qPCR primers** | **Sequence (5' to 3')** | **Product size** | **Annealing temperature (°C)** |
| --- | --- | --- | --- |
| FAT1 F | TTCAAAATAGGTGAAGAGACAGGTG | 170bp | 59 |
| FAT1 R | TTGTGATGAGACCTGTTTTAGGATG |  |  |
|  |  |  |  |
| 18S F | GTAACCCGTTGAACCCCATT | 160bp | 59 |
| 18S R | CCATCCAATCGGTAGTAGCG |  |  |
|  |  |  |  |
| RelA F | CTGCATCCACAGTTTCCAGAAC | 260bp | 60 |
| RelA R | TCTTGAGCTCGGCAGTGTT |  |  |
|  |  |  |  |
| **ChIP RelA primers** | **Sequence (5' to 3')** | **Product size** | **Annealing temperature (°C)** |
| FAT1 CHIPF | CAGGGACTGGGGCTCTTTG | 190 | 63 |
| FAT1 CHIPR | GCCTCACGCTCCCCGAG |  |  |
|  |  |  |  |
| **SDM RelA primer** | **Sequence (5' to 3')** | **Product size** | **Annealing temperature (°C)** |
| RelA SDM F | GGCAGCCGAATTCAGTGGGCTCGAGTTTCCGCGC | 6000bp | 62 |
| RelA SDM R | ACTCGAGCCCACTGAATTCGGCTGCCGGCTCTGGG |  |  |
